# KDeep: a new memory-efficient data extraction method for accurately predicting DNA/RNA transcription factor binding sites

**DOI:** 10.1186/s12967-023-04593-7

**Published:** 2023-10-16

**Authors:** Saeedeh Akbari Rokn Abadi, SeyedehFatemeh Tabatabaei, Somayyeh Koohi

**Affiliations:** Department of Computer Engineering, Sharif University of Technology, Tehran, Iran

**Keywords:** Transcription factor, Binding site, k-mer, CNN, LSTM, Interpretability

## Abstract

This paper addresses the crucial task of identifying DNA/RNA binding sites, which has implications in drug/vaccine design, protein engineering, and cancer research. Existing methods utilize complex neural network structures, diverse input types, and machine learning techniques for feature extraction. However, the growing volume of sequences poses processing challenges. This study introduces KDeep, employing a CNN-LSTM architecture with a novel encoding method called 2Lk. 2Lk enhances prediction accuracy, reduces memory consumption by up to 84%, reduces trainable parameters, and improves interpretability by approximately 79% compared to state-of-the-art approaches. KDeep offers a promising solution for accurate and efficient binding site prediction.

**Supplementary Information:**

The online version contains supplementary material available at 10.1186/s12967-023-04593-7.

## Background

DNA and RNA binding proteins play a vital role in gene regulation, including alternative splicing, transcription, and translation. According to these important roles, any disorder in their function can increase the risk of complex diseases [1–3]. RNA-Binding Proteins (RBPs) and DNA-binding proteins, which include bound proteins to double or single-stranded RNA/DNA recognize short motifs within the RNA/DNA, as the binding sites to bind them [4]. Therefore, finding these motifs is crucial since they provide an explanation for the molecules binding and allow for generalized statement for binding other sequences to the related proteins [5, 6].

Due to the limitations of laboratory methods to identify these motifs (e.g. being expensive, time-consuming, and equipment dependent), as well as the rapid growth of strands and improvement in processing capability in recent years, there is a strong desire to alternate them with the computational approaches [7–10], especially Machine Learning-based (ML) methods[11, 12]. However, the binding sites found in the labs only partially overlap with the motifs identified by the alignment approaches, which complicates prediction of these patterns using computational methods [13]. It is worth mentioning that a combination of physical, chemical, and structural factors evolves a motif into a location where proteins may bind to DNA or RNA, and so, it is difficult to extract [4, 14, 15]. Of course, studies have revealed that the majority of these features are connected to the strands’ context directly surrounding the motif. However, some studies, such as deepnet-rbp [16] and DeepRKE [17], take advantage of other features, such as secondary and tertiary RNA structures, to increase the accuracy of binding site identification.

Aforementioned issues motivated the recent studies to propose deep neural network-based architectures, which mainly include three models CNN, LSTM, and CNN-LSTM [12]. According to these studies, CNN-based networks, such as DeepBind [18] and DeepSea [19], were able to achieve acceptable interpretability. On the other hand, recently proposed LSTM-based architectures, such as DanQ [20], DeepRKE [17], DeepSite [21], DeepDRBP-2L [22], and WSCNNLSTM [23] conclude that taking advantages of LSTMs as the memory-driven unit [24] in a CNN-based network increases tool’s accuracy, but it reduces the output’s interpretability. Of course, in general, including extra layers to the learning model, at the cost of increasing complexity, can increase the prediction accuracy [12]. Moreover, it should be noted that all aforementioned tools require a pre-processing step to generate customized input for them, and so, this phase can have a significant impact on the tools' performance [12, 13, 15]. While less attention is paid to this area in the design of binding sites prediction tools for transcription factors, designing complex learning architectures and including auxiliary data of DNA/RNA sequences, such as secondary structures, leads to more complicated process [25, 26]. It is worth mentioning that there exist two well-known encoding methods, i.e. One-hot and Word2vec [27, 28], that address the preprocessing step; specifically, the One-hot encoding which is used by various methods, such as Deepbind, iDeepS [29], DanQ, DeepSea, simplifies the interpretation of the model due to its simple encoding algorithm. On the other hand, Word2vec, as another well-known encoding method, results in more accurate prediction, compared to the One-hot encoding method, in various tools, such as iDeepV [30], DeepRKE [17], KEGRU [31], and DeepRam [13]. However, the latter accuracy improvement comes at the cost of heavy learning process, as well as reduced output’s interpretability.

Summarizing above discussion, we can conclude that taking advantages of a good feature extraction method for the pre-processing step can improve the predictor accuracy, the model’s complexity, and last but not the least, the resource requirement. Therefore, in this study, by addressing the encoding phase, we have developed a novel encoding method, named 2Lk, to affect many features of the binding site predictor. Specifically, the proposed encoding method takes advantages of 2 Levels of k-mer: the first level is based on the k-mer sliding window and the second one is based on the k-mer representation by FCGR. As a computational algorithm, unlike the word2vec, 2Lk does not require a training phase, and also, needs less runtime and computational resources than the learning-based encoding algorithms. Additionally, compared to the alternative learning-based encoding methods, such as word2vec, our encoding method produces smaller but more informative vectors, and so, reduces the total number of trainable parameters of protein binding side predictors. The latter achievement can help the main application by reducing resource consumption, like memory usage, power consumption, execution time, and CPU/GPU utilization, due to the fact that 2Lk is not based on machine learning. On the other hand, the proposed encoding method can enhance the efficiency of binding site predictors in comparison to the simple encoding methods, like one-hot, and complex encoding methods, like word2vec, by preparing additional informative features of strands. Finally, to propose a novel binding site predictor, we use the hybrid CNN-LSTM architecture with the attention layer as the predictor unit. It should be noted that the attention layer increases the predictor accuracy, since it can determine which part of the input is essential to generate the outputs, so we can find motif’s range of position. Performing the interpretation task by transforming convolution kernels to motifs is another crucial subject for TF binding site predictor taken into consideration in this work.

As the comparative study, to assess 2Lk and demonstrate the capability of this encoding method, we compared the proposed predictor tool with some well-known and cutting-edge tools using three benchmark DNA/RNA datasets. According to these comparisons, we can conclude that 2Lk can outperform the alternative methods in various aspects, as listed below:

- Extracting informative features from the input strands with no learning phase.
- Improving memory usage, compared to the state-of-art methods, like word2vec encoding, as well as those methods taking extra information as input, like secondary structure, tertiary structure, and reverse complementary of strands [32].
- Reducing total number of trainable parameters of protein binding side predictors, and so improving the resource utilization, as well as the execution time.
- Improving accuracy of prediction and motif extraction, compared to the state-of-art methods.
- Improving interpretability, compared to the alternative methods, such as word2vec and one-hot.

## Result

Since the suggested KDeep is focused on optimizing the preprocessing step and encoding, we compare it against numerous state-of-the-art approaches from diverse classes. These classes include different encoding techniques (one-hot and word2vec), input types (only primary strands, both primary strand and the secondary structure, or all three primary, secondary, and tertiary structure RNAs), and predictor architectures (CNN and LSTM-CNN with various levels of complexity). Table 1 includes detailed specifications of the compared approaches.

**Table 1:** Specifications of the studied tools; KDeep, KDeep + , DeepRKE-, DeepRKE + , GraphProt, DeepBind, iDeepS, DanQ, iDeepV, deepnet-rbp, mmCNN

Methods	Strand type	Input type	Encoding method	Predictor architecture
KDeep	RNA/DNA	Primary	2Lk	CNN + LSTM
KDeep +	DNA	Primary	2Lk	CNN + LSTM + Attention
DeepRKE-	RNA	Primary	Word2vec	CNN + LSTM
DeepRKE +	RNA	Primary + secondary	Word2vec	CNN + LSTM
GraphProt	RNA	Primary + secondary	Graph	SVM
DeepBind	DNA/RNA	Primary	One-hot	CNN
iDeepS	RNA	Primary + secondary	One-hot	CNN + LSTM
DanQ	DNA	Primary	One-hot	CNN + LSTM
iDeepV	RNA	Primary	Word2vec	CNN
deepnet-rbp	RNA	Primary + secondary + tertiary	(Primary & Secondary) encode by replicated software model RNA, tertiary structure encoded by RNA 3D Motif	CNN
mmCNN	RNA	Primary + secondary + tertiary	Primary (one-hot) Secondary (Structure Probability Matrix)	CNN

Our simulation scenarios are categorized in two classes: a) evaluating the impact of encoding techniques (2Lk (3, 2), 2Lk (3, 3), word2vec (50), word2vec (100), and one-hot) on the prediction accuracy, considering fix predictor architecture, and b) evaluating the prediction accuracy achieved by KDeep tool, against the cutting-edge techniques, for RNA and DNA datasets.

Performance is assessed using two of the most popular metrics: Area Under the precision recall curve (auPRC) and Area Under the Receiver Operating Characteristic Curve (auROC). It should be noted that the degree or measure of separability is represented by the auROC, as a performance metric for classification issues at various threshold levels. On the other hand, auPRC is almost the average of precision scores calculated for each recall threshold. This metric is used for performance measurement, particularly in the case that the dataset is extremely unbalanced and/or the positive class is prioritized. auPRC considers the positive predictive value PPV and the true positive rate TPR, whereas auROC looks at the true positive rate TPR and the false positive rate FPR. In this manner, we utilize both auPRC and auROC to evaluate two different classification scenarios: a) the positive class is considered more important, and b) both positive and negative classes are considered important. Of course, similar to the alternative studies, we also use auROC for RBP datasets and both auRPC and auROC for DNA strands.

### Investigating the impact of sequence encoding method on the predictor performance

There are many factors that affect the effectiveness of DNA/RNA binding site predictions, including architectures, input types, and strand representation techniques. So, to clarify the impact of strands' features on the methods efficiency, in this assessment, we employ the CNN-LSTM architecture, as shown in Fig. 7a, that calls KDeep as the fixed architecture and apply it to all popular strand encoding methods. In order to evaluate the impact of 2Lk encoding, we choose one-hot and word2vec methods with 50 and 100 features, respectively, for 3mers, and compare them with two versions of 2Lk with size k = 2 and k = 3. The dataset used for these assessments is RBP-31. Finally, these comparisons are performed in terms of predictors’ performance and their resources consumption.

### Predictors’ performance

**Fig. 1:**
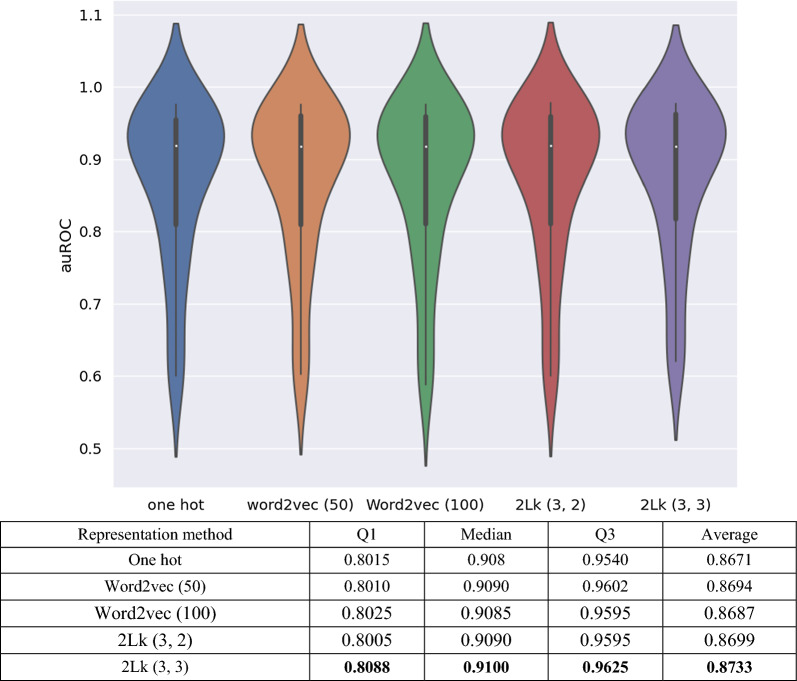
Comparing auROC distribution of five encodings methods: one-hot, word2vec (50), word2vec (100), 2Lk (3, 2), and 2Lk (3, 3) with fixed predictor architecture KDeep

**Fig. 2:**
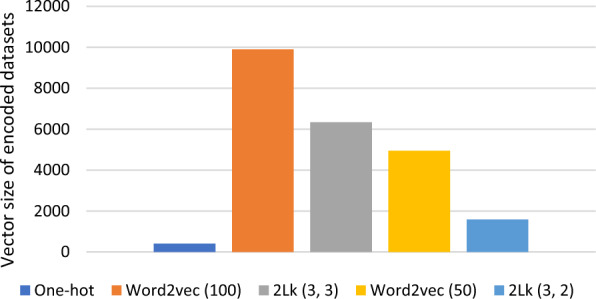
Vector size of encoded datasets for 5 cases of sequence encoding methods

Figures 1 and 2 depict the simulation results by auROC distribution plots for all 31 cases and the total vector size of encoded datasets by each method. According to Fig. 1, any information of local patterns, such as k-mers information prepared by 2Lk and word2vec methods, can improve predictors' accuracy, as compared with simple encoding methods, like one-hot. However, considering k-mer-based methods, complicated approaches, such as word2vec, may not always offer further improvement. Indeed, the simulation results demonstrate that the proposed computational approach, 2Lk (3, 3), achieves higher auROC to that of the learning-based method, word2vec (50). All of these enhancements achieved by 2Lk come with reduced represented vector sizes (as shown in Fig. 2) and higher interpretability, compared to the word2vec, as a learning-based encoding method.

In this assessment, we have compared each of the two k-mer-based encoding methods for two sizes. Examining the impact of the size of k_2_ in 2Lk, Fig. 1 shows that the average value of auROC for the size of k_2_ = 3 improves, compared to that of k_2_ = 2, by more than 0.003. Of course, this improvement is achieved at the cost of larger encoding size which can be seen in Fig. 2. According to Fig. 1, although previous studies concluded that word2vec achieves the best accuracy with a vector length of 100 for each word [33], as our comparative studies confirm, KDeep with the word length of 50 results in about 0.001 higher average accuracy. This improvement is achieved despite the fact that word2vec with the size of 100 generates larger encoded dataset, according to Fig. 2. Detailed simulation results various methods are provided in Additional file 2.

Finally, according to Fig. 1, we can conclude that 2Lk (3, 2) with small vector size of 16 (= 4 × 4) for each k-mer can achieve a higher average auROC, compared to the learning-based method word2vec (50). Moreover, according to Fig. 2, 2Lk reduces vector size, so it improves the memory consumption and execution speed, especially for high-volume datasets with large number of hyper-parameters, where the resource consumption is challenging.

In addition to these results, we also calculated the number of trainable parameters of the five compared representation methods, which can be seen in Additional file 3: Fig. S4, according to which, in general, 2Lk has improved the number of trainable parameters, compared to one-hot and word2vec methods.

### Hardware resources’ utilization

One of the important aspects affecting the hardware system's performance and resources utilization in ML-based approaches is the encoding technique. In this regard, as a comparative study, we used Weights & Biases [34] to monitor the hardware resources utilized by five different encoding methods adopted in the KDeep model, including one-hot, word2vec (50), word2vec (100), 2Lk (3, 2), and 2Lk (3, 3). Finally, for the five aforementioned methods, we examined 14 different metrics evaluating usages of GPU, CPU, disk, memory, and network states, as reported in section “Hardware resources” of the Additional file 3. We performed this analysis on one of 31 and one of 24 sub-datasets of RBP 31 and RBP 24, respectively. The trend of the results is consistent in both samples. However, since the sequences in RBP 24 are longer, the advantage of the KDeep method is more pronounced. Therefore, we have reported the results for both cases in Additional file 3: Figs. S2, S3, respectively for RBP-31 and RBP-24.

As shown in all part of Additional file 3: Fig. S2, it can be observed that both word2vec (50) and word2vec (100) use highest system times, and even, with their highest power consumption fallen within the bounds of other approaches, they ultimately result in worse energy conditions. For instance, according to the graph of GPU Power Usage (Additional file 3: Fig. S2a), word2vec (50) and word2vec (100) use more energy compared to the alternative methods, or they maintain the GPU's temperature at a high level for a longer period of time, according to the graph of GPU Temp (Additional file 3: Fig. S2e). A similar behavior happens for their Process Memory In Use (Additional file 3: Fig. S2k). Of course, according to Additional file 3: Fig. S2n, they lead to more CPU Utilization, compared to the alternative methods.

Another observation that can be drawn from these graphs is that in addition to the 2Lk (3, 3) has lower memory consumption for both the GPU and system (Additional file 3: Fig. S2c–m, respectively), this utilization occurs for a short period of time. Indeed, the 2Lk (3, 2) method behaves exactly similar to the one-hot method, which is thought to be the best method in terms of resource utilization. Meanwhile, 2Lk (3, 2) leads to higher accuracy than one-hot, as was discussed in the preceding section.

The process for the RBP 24 dataset follows the same steps. It should be noted that the execution conditions are the same for all encoding methods in order to have a fair comparison, these conditions are listed in Additional file 3: Table S4. One of our goals in designing the 2LK method is to improve memory consumption. Therefore, we will discuss this in more detail below. As shown in Additional file 3: Fig. S3c, word2vec (100) has the highest GPU memory consumption, while one hot and 2LK (3, 2) have the lowest. Since the length of the DNA strand in this dataset is 375, applying word2vec (100) encoding will result in a vector size of 373 × 100. The vector size for 2LK (3, 2) will be 373 × 16, and for one hot, it will be 375 × 4. Therefore, it is expected that word2vec (100) will have the highest memory consumption. Actually, for longer string lengths, such as in datasets like DeepSea with a length of 1000 or in cases like enhancer sequences, GPU memory consumption becomes even more critical. Note that encoding operations are performed on the CPU for both word2vec (100) and word2vec (50) methods from 0 to about 200 s, which is why memory consumption is close to zero during this time.

Additional file 3: Fig. S3f displays the GPU utilization, indicating that the highest usage values are associated with word2vec (100), 2LK (3, 3), word2vec (50), 2LK (3, 2), and one hot, respectively. These values are determined based on the total memory consumption, power, and temperature, highlighting the significance of encoding length in GPU utilization. This importance is particularly noteworthy for datasets with high train data values, such as the DeepSea dataset with 4,400,000 train samples.

Additional file 3: Fig. S3m illustrates the CPU memory consumption, with the highest value associated with 2LK (3, 3), while the other cases are closely grouped. The difference in CPU memory consumption is more pronounced in datasets with a high number of train samples. Additional file 3: Fig. S3n displays the CPU utilization, with the highest CPU consumption related to word2vec (100) and word2vec (50). The increase in execution time is attributed to the type of machine learning-based encoding and the number of training samples. The encoding process becomes more time-consuming with an increase in the number of train samples. However, in the 2LK (3, 2) and 2LK (3, 3) methods, the encoding part is only calculated for all possible 3-length nucleotide and N-letter cases, which becomes 125 cases and is completely independent of the number of train samples. Therefore, it has a high speed and consumes fewer resources for encoding. Considering the accuracy and resource consumption, it can be concluded that our method exhibits good performance. It should be noted, to demonstrate that the 2LK method improves both resource consumption and efficiency for the RBP-24 dataset, we have included Additional file 3: Table S3 specifically for this dataset performance.

### Comparing the binding site predictors’ performance for RNA datasets

**Fig. 3:**
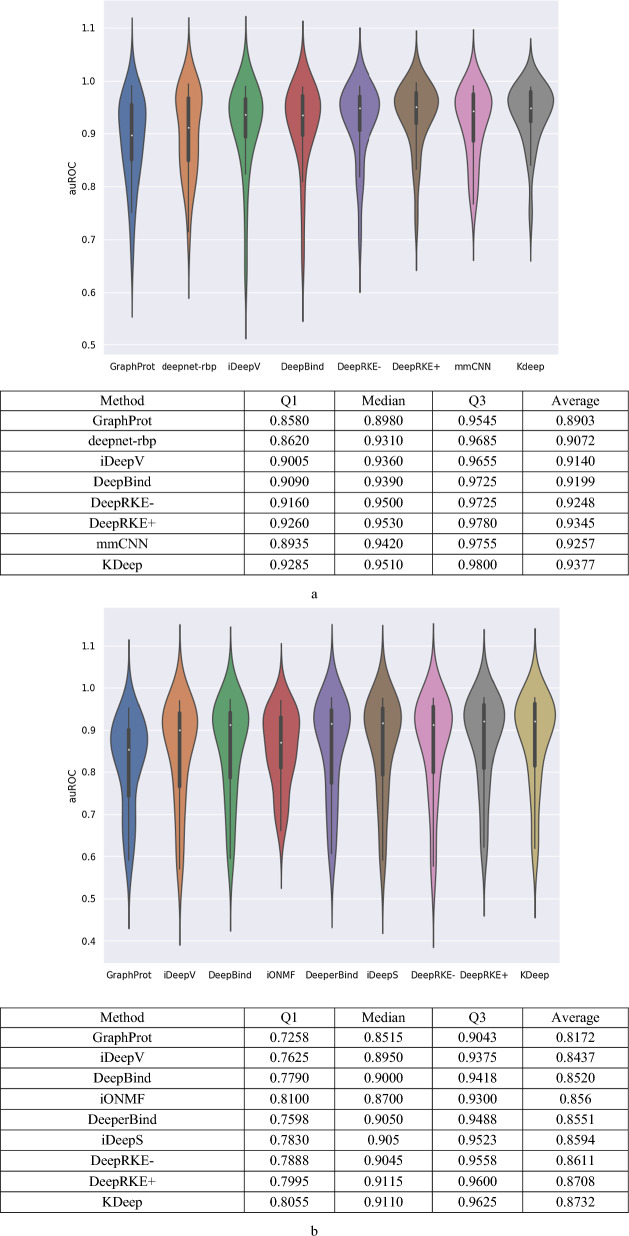
**a** Comparing auROC distribution of eight encodings methods for RBP-24, **b** Comparing auROC distribution of eight encodings methods for RBP-31

To evaluate KDeep for predicting RBP sites, two datasets are considered, RBP-24 with variable length strands and RBP-31 with fixed length strands. Evaluating RBP-24 datasets, we compare classification performance of KDeep with other 7 RBP-site predictors, including DeepRKE-, DeepRKE + , GraphProt, deepnet-rbp, DeepBind, mmCNN [35] and iDeepV. Figure 3a demonstrates the auROC distribution for the RBP-24 dataset, as well as the distribution of 24 auROC values for each RBP achieved by each method. As shown in Fig. 3a, for RBP-24, KDeep achieved the average auROC of 0.9377 which confirms its outperformance over the alternative methods. In more details, KDeep obtains the best auROC values for 12 out of total 24 sets of RBPs, while for the others, it is among the best three methods. For example, for IGF2BP1-3 PAR, KDeep obtains the auROC value of 0.958, as the best method among all studies approaches. Specifically, its obtained auROC value is higher by more than 1.59% against that of the DeepRKE + (i.e. 0.943), and also, it increases auROC value by 2.57%, compared to the DeepRKE (i.e. 0.934). Detailed results for each of these 24 experiences are reported in Additional file 4.

As another RBP dataset, we consider RBP-31 with fixed length strands to evaluate KDeep against other 7 RBP-site predictors, including DeepRKE-, DeepRKE + , GraphProt, iDeepV, DeepBind, iONMF and iDeepS. Considering 31 experiences of RBP, the average auROC value achieved by KDeep is 0.876, which confirms its better overall performance compared to the alternative methods. In more details, KDeep obtains the best auROC values for 19 out of total 31 sets of RBPs, while for the remaining 12 instances, its auROC values are among the top two values with minimal variation. Figure 3b demonstrates the distribution of auROC values, as well as the corresponding average values for the RBP-31 dataset. Detailed results for each of these 31 experiences are reported in Additional file 5.

As a key point, it should be noted that while KDeep is much simpler, it outperforms the current state-of-the-art method DeepRKE + and gets an average auROC value of 0.8732 for RBP-31 dataset and 0.9377 for RBP-24 dataset. In more details, KDeep, unlike the DeepRKE + , does not use the secondary structure as its input, and so, it uses a smaller and simpler input vector. Utilizing the primary strand as the only input vector, KDeep takes advantages of single-layer CNN, while DeepRKE + uses three layers of CNN which lead to a complex learning model. Moreover, utilizing word2vec, as a learning-based encoding method, DeepRKE + requires more computational resources and processing time, compared to our proposed encoding method. Specifically, the adopted word2vec encoding method generates input vectors of size 100 entries for each word in DeepRKE + , while the length of the input vector for each word in 2Lk encoding used by KDeep is 64 (= 8 × 8). In this manner, as shown in Fig. 2, KDeep leads to less memory usage for large datasets, compared to the state-of-the-art method DeepRKE + . As the last but not the least advantage, due to the complexity of the learning model and the encoding method of DeepRKE + , interpretability is much more difficult to achieve, unlike the KDeep. Due to the later reason, DeepRKE + has not addressed interpretability and motif extraction.

As another version of DeepRKE + [17], DeepRKE- [17] just uses primary strand of RNA as the input vector, and so, its architecture is similar to that of the KDeep. However, as its main difference from KDeep, is adopts word2vec encoding method. It is worth noting that according to the different encoding method, KDeep can improve auROC of RBP-24 and RBP-31 by more than 1.4% and 1.5%, respectively, compared to the DeepRKE-. This outperformance is despite the fact that 2Lk in KDeep does not require a learning-based method to generate the input vector, and it also reduces the memory usage of the encoded strands, as shown in Fig. 2.

### Comparing binding site predictors’ performance for DNA datasets

**Fig. 4:**
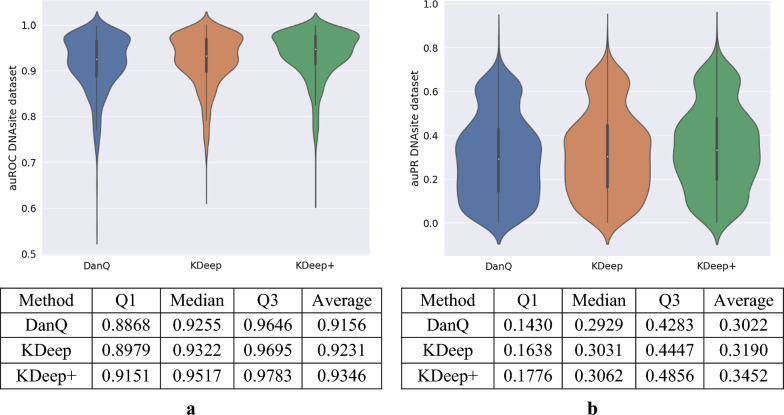
Comparing auROC and auPR distribution of three binding site predictor methods for DNAsite dataset; **a** auROC for DNAsite dataset, **b** auPR for DNAsite dataset

**Fig. 5:**
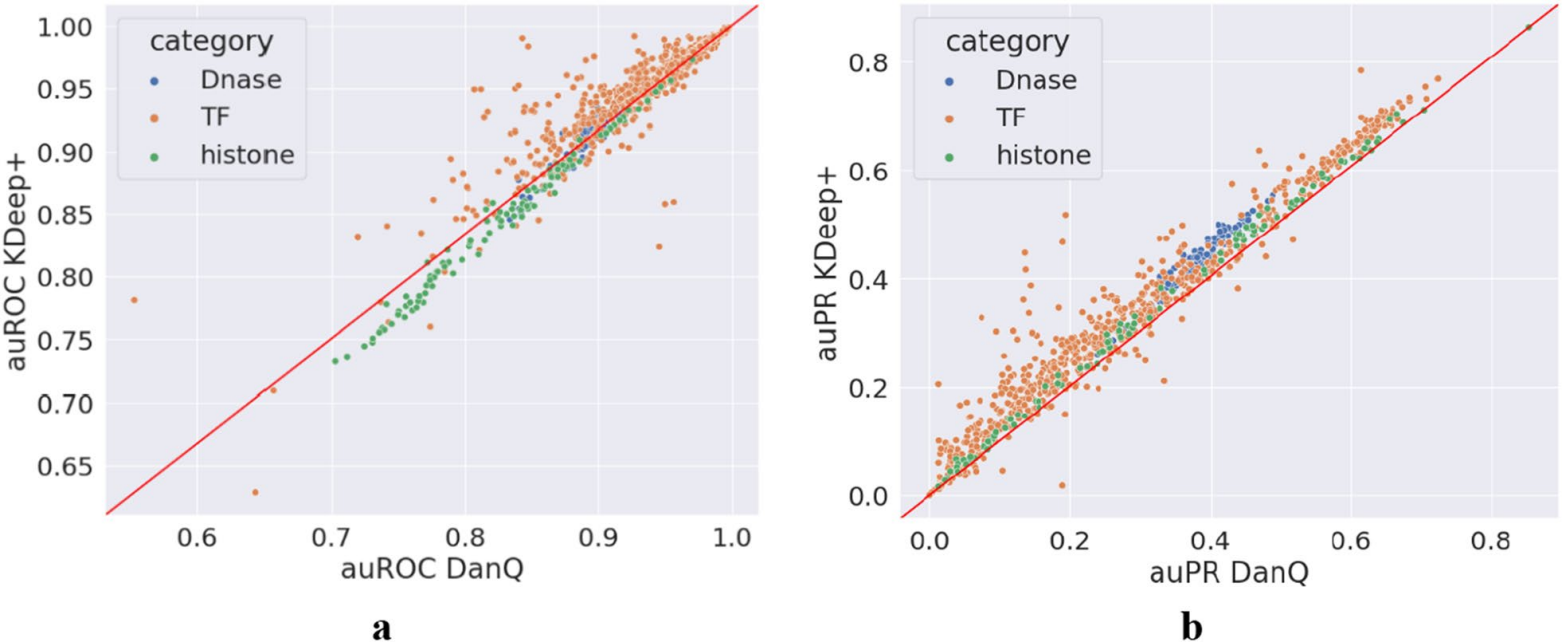
Scatter plots of auROC and auPR scores of DanQ and KDeep + . The x-axis corresponds to the auROC/auPR scores of DanQ and the y-axis corresponds to the auROC/auPR scores of KDeep + ; **a** auROC scores for 919 targets and three types of data. KDeep + outperforms DanQ for 95.9% of the 919 targets, **b** Scatter plot of the auPR scores for 919 targets and three types of data. KDeep + outperforms DanQ for 94.8% of the 919 targets

As previously indicated, KDeep can also be applied on DNA sequences for finding various types of protein binding sites in these sequences. For this purpose, DNAsite dataset, as a popular dataset in this field, is selected to evaluate KDeep. As previously mentioned, DNAsite dataset includes 919 classes among DNase-seq, Transcription-factor, and Histone-mark data in various cell types. In this assessment, we examine the capability of KDeep in two versions (i.e. with and without attention layer), compared to the DanQ [20] which is a popular method for DNA sequences based on one-hot encoding, and report auROC, auPR, and scatter plots, as shown in Figs. 4 and 5, respectively. Since DNAsite dataset consists of three types of data, the metrics are reported as the total average values for all types, as well as the separate value for each of three types of data. Of course, information about these three types of data is accessible in section “KDeep’s performance for DNA datasets” of the Additional file 3: Fig. S3. In addition, the auROC and auPR values of each 919 targets for DanQ, KDeep and KDeep + are accessible in Additional file 1.

Based on auROC and auPR, the prediction accuracy of KDeep and KDeep + is higher than that of DanQ, on average. These improvements can be seen in Fig. 4 as well as scatter plots provided in Fig. 5. Of course, KDeep + has made improvements by a greater margin over the DanQ, so that KDeep + outperforms DanQ for 95.9% of the 919 targets in terms of auROC, and 94.8% of the 919 targets in terms of auPR. As another point that can be established from this data, it is worth noting that these improvements are different in the three categories of DNAsite datasets, in a way that more improvement is obtained for Dnase data and less improvement for Histon data (Additional file 3: Fig. S4e, f). According to Fig. 5, KDeep + has a lower auROC but a better auPR than DanQ in the histone category. This suggests that while KDeep + may not be as effective at distinguishing between positive and negative labels in histone sequences as DanQ, it may be better at identifying true positive labels. Related to the imbalanced nature of the histone dataset, there is a higher proportion of positive labels compared to negative labels (non-histon labels). In such cases, a model that is better at identifying true positive labels (as measured by auPR) may be more useful than a model that is better at distinguishing between positive and negative labels (as measured by auROC). Related to the specific architecture of KDeep + and DanQ, it seems each model is suited for different types of sequence data. For example, KDeep + may be better at capturing certain features or patterns in histone sequences that are important for predicting positive labels, while DanQ may be better at capturing other features or patterns that are important for distinguishing between positive and negative labels. Overall, the fact that KDeep + has a better auPR but a lower auROC than DanQ in the histone category suggests that it may be a useful tool for identifying true positive labels in imbalanced datasets, but further investigation is needed to fully understand the reasons for this difference in performance which we will deal with in future works.

Of course, improvement in prediction accuracy becomes more important when it is accompanied by improvement in the number of trainable parameters. In addition to increasing the accuracy, KDeep has also reduced the number of trainable parameters of the model as the result of its novel encoding method. The latter achievement improves the resource consumption, as well as the training speed. Specifically, KDeep and DanQ take about 3000 s and 3957 s for each epoch, respectively. On the other hand, it should be noted that although KDeep + reduces the number of trainable parameters, but the learning time has increased to 5872 s for each epoch, as the results of adopting extra attention layer. Total numbers of trainable parameters for DanQ and our methods are shown in Additional file 3: Fig. S5.

### Visualization

To find binding sites patterns, we use the visualization technique for both DNA and RNA sequence types. However, it should be noted that due to the lack of interpretability analysis of alternative methods for RNA sequences, comparing KDeep’s interpretability with them may face some challenges. However, KDeep's results for RNA are thoroughly given in Section “Visualization” of the Additional file 3: Fig. S6.

Thanks to the more detailed reports on interpretability analysis of various methods in the case of input DNA sequences, as follows, we provide comparative study addressing interpretability of KDeep against that of alternative methods in more details. Since we have 320 kernels in the first convolution layer of KDeep for DNA learning, we were able to extract all 320 motifs out of 320 motifs, among which 181 and 113 known motifs were significantly matched with E-Value less than 0.01 by KDeep and KDeep + , respectively. Some of the detected DNA motifs by KDeep and KDeep + and also the heatmap of output score of the attention layer of KDeep + for a number of samples with the ability of TF binding are shown in Additional file 3: Figs. S7, S8, and S9, respectively, in Section “Visualization” of Additional file 3. In general, according to these results, KDeep (with or without the attention layer) is able to obtain better interpretability, compared to DanQ, noting that DanQ can only obtain 101 significantly matched known motifs, out of 320 motifs, with E-Value less than 0.01. Based on these results, it can be concluded that the KDeep and KDeep + methods outperform the DanQ method, in reporting motifs, by more than 79% and 11%, respectively. Moreover, according to these results, we can conclude that KDeep, compared to the KDeep + , can extract larger number of motifs taking advantages of CNN. Of course, the attention layer embedded in KDeep + facilitates specifying the range of motifs within the sequences, as well as increases the prediction accuracy.

## Discussion

In this work, we proposed KDeep and KDeep + , as the k-mer based tools that predict binding site of DNA/RNA to proteins. Our proposed encoding method, 2Lk, adopted in both KDeep and KDeep + , extracts more understandable machine learning information from the strands, compared to the alternative encoding methods. Specifically, the kernels in the CNN layer act as a scanner to capture motif information from the input DNA/RNA sequences, while the BiLSTM layer enables learning the regulatory grammars of the motifs extracted by the CNN layer. Moreover, we took advantages of the attention mechanism in KDeep + to highly focus on the important locations within the extracted motifs to predict DNA binding to the proteins, which in turns increased the prediction accuracy. In this manner, by enhancing the encoding method and including the attention mechanism, we could significantly improve the current state-of-art methods.

We used three famous datasets to evaluate KDeep and KDeep + and compare them with the state-of-art methods for DNA/RNA binding site prediction. Based on the results for RNA datasets, KDeep compared to the state-of-art methods offers several advantages, as increases the prediction accuracy, (2) improves the resources consumption, such as RAM usage, by taking advantages of not learning-based encoding method as well as reducing coding vector length, (3) reduces the number of trainable parameters of the model, which in turn reduces the learning time, and finally, (4) provides TF binding site prediction only based on the primary structure of the strands, which in turn simplifies the learning model, and as a result, leads to reduced resources consumption, and improved prediction performance. KDeep + outperformed DanQ for 95.9% of the 919 targets in terms of auROC values and 94.8% of the 919 targets in terms of auPR values for DNA datasets. For 407 out of 919 targets, KDeep + achieved average auROC values 2.2% higher than those of DanQ. Moreover, KDeep + achieved significantly higher auPR scores, in average, compared to DanQ. Specifically, DanQ's average auPR is 0.302, while KDeep + can achieve average auPR value of 0.345, which shows increment by 14.2%.

To extract the RNA/DNA motifs, we used convolution layer filters According to various evaluation results, we concluded that the proposed motif extraction model outperforms the complex models with several CNN layers, which take the primary and secondary structures, as well as the complement of the strand as input. Moreover, KDeep and KDeep + simplify the motif extraction, compared to the word2vec encoding, as a learning-based method. On the other hand, by including the attention layer in KDeep + , we facilitate finding the range of motif positions within the DNA strand. Finally, KDeep and KDeep + were able to extract larger number of DNA motifs, as the significantly matched known motifs, out of 320 motifs, with E-Value less than 0.01.

Although KDeep has many advantages, there are still challenges in this area. For example, we plan to use and code the datasets including sequences with very different lengths in the near future. Moreover, while in our interpretability method, the effect of the LSTM layer has been neglected, we will try to provide a better interpretable method to extract motifs.

## Conclusion

In conclusion, this study presents KDeep and KDeep + , innovative tools for predicting DNA/RNA binding sites. Leveraging a CNN-LSTM architecture and the 2Lk encoding method, these models demonstrate improved prediction accuracy and resource efficiency compared to existing methods. By capturing important motifs and regulatory grammars within DNA/RNA sequences, KDeep and KDeep + offer valuable insights into biological processes and facilitate advancements in drug design, protein engineering, and cancer research.

The evaluation results on RNA and DNA datasets highlight the superiority of KDeep and KDeep + over state-of-the-art approaches. KDeep achieves higher accuracy while reducing resource consumption, demonstrating its potential for large-scale sequence analysis. Moreover, KDeep + outperforms the widely used method, DanQ, in terms of auROC and auPR values, showcasing its effectiveness in predicting DNA binding to proteins. These models not only enhance prediction performance but also offer interpretability through the identification of important motifs and their positions within the DNA strand. Future directions include addressing challenges related to diverse sequence lengths and further improving the interpretability of motif extraction methods.

## Method

### Datasets

We employ three large benchmark datasets, as listed in Table 2, to examine a variety of binding situations, noting that KDeep is designed to predict the protein binding sites to both DNA and RNA strand types. The RBP-24 dataset [36], which consists of 24 sets of CLIP-seq [37] and 9 sets of RNAcompete data, is one of our three datasets and is considered to support RBP prediction from various forms of high-throughput experimental data, such as CLIP-seq and RNAcompete. RNA strands in RBP-24, which was introduced by GraphProt, vary in length from 150 to 375 nucleotides. Of course, it should be noted that each of the 24 cases in this dataset contains almost an equal number of positive and negative samples, making it a balanced dataset. The RBP-31 dataset, which consists of fixed-length RNA sequences with 101 nucleotides, is our next dataset including RNA strands. The CLIP-seq data in this dataset, as is collected in iONMF [38], is made up of 19 proteins and 31 experiments, and its annotations are based on the human assembly hg19. Each nucleotide, contained in clusters of interaction sites obtained from CLIP-seq, is regarded as a binding site, as stated in iONMF. The final point to note about this dataset is that it is almost unbalanced. Out of 30,000 training samples, only 6,000 are positive labels, while the remaining are negative labels. Similarly, out of 10,000 test samples, only 2,000 samples are positive labels, while the rest are negative. It should be mentioned that both datasets include train and test data, as listed in Table 2. The third popular dataset includes DNA strand binding sites and is introduced by the Deepsea framework. For this dataset, the intersection of 919 ChIP-seq and DNase-seq peak set [39] (includes 125 DNase features, 690 TF features, and 104 Hsiton-markfeatures), from uniformly treated releases of ENCODE and Roadmap Epigenomics data, results in the computation of the targets. Then, the 1000 bp-long DNA strands from the human GRCh37 reference genome are selected as the samples including 200 bps labeled sequences with 919 target chromatin features. This dataset consists of 8000 validation samples, 4,400,000 training samples, and 113,756 test samples. Section “Datasets” in the Additional file 3 explores these triple datasets in more details.

**Table 2:** Specifications of CLIP-seq and RNAcompete

#	Dataset-Name	Length of strands	Length of label-vector	#Train-samples	#Test-samples
1	RBP-24	150–375	1	Different	1000
2	RBP-31	101	1	30000	10000
3	DNAsite dataset	1000	919	4400000	113756

### Preprocessing method

**Fig. 6:**
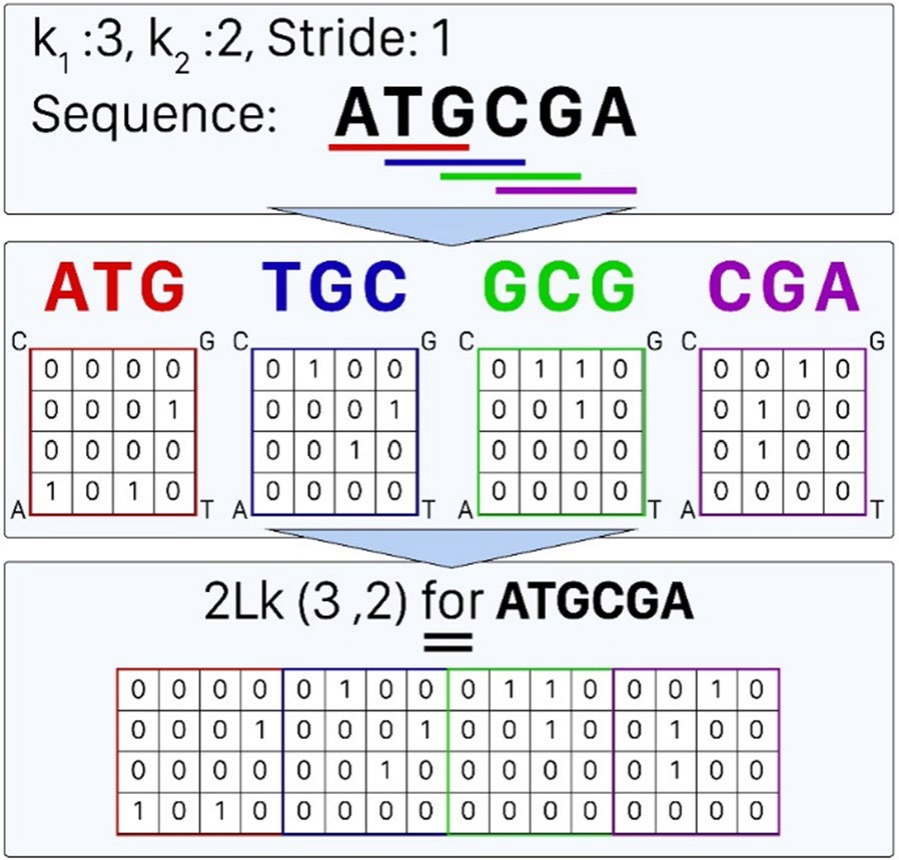
Algorithm’s steps of the proposed encoding method, 2Lk, for sample sequence ATGCGA with k_1_ = 3, stride = 1, k_2_ = 2

Noting that DNA/RNA binding sites prediction is primarily a motif-finding application, preserving the order of nucleotides throughout encoding process is also required. There are two ways to approach this goal, nucleotide-to-nucleotide encoding and k-mer-based encoding. We consider the later form of encoding since it is more informative due to considering neighbor nucleotides into account. To obtain k-mers, a sliding window traverses the sequences in step sizes of one, as shown in Fig. 6. Once k-mers are obtained for subsequent windows, they are vectorized and combined to encode the complete strand. As a key advantage of our encoding method, 2Lk, it adopts the FCGR method (more details about this algorithm are described in section “FCGR algorithm” of the Additional file 3) to encode these k-mers. In fact, FCGR is a rough approach to profile k-mers of a strand in a matrix representing the number of occurrences of all possible k-mers of the input sequence. So it generates a matrix with 4^ k^ entries, each of which representing a specific k-mer’s frequency. In this manner, our encoding method generates a vector of $(L-{k}_{1}+1)\times {4}^{{k}_{2}}$ entries for each DNA/RNA strand, where L is length of strand, k_1_ is the size of sliding window, and k_2_ is the size of FCGR matrix. To represent the proposed encoding method, we use 2Lk(k_1_, k_2_) notation. For more clarity, Fig. 6 provides an overview of a sample 2Lk(k_1_, k_2_).

### Predictor model architecture

**Fig. 7:**
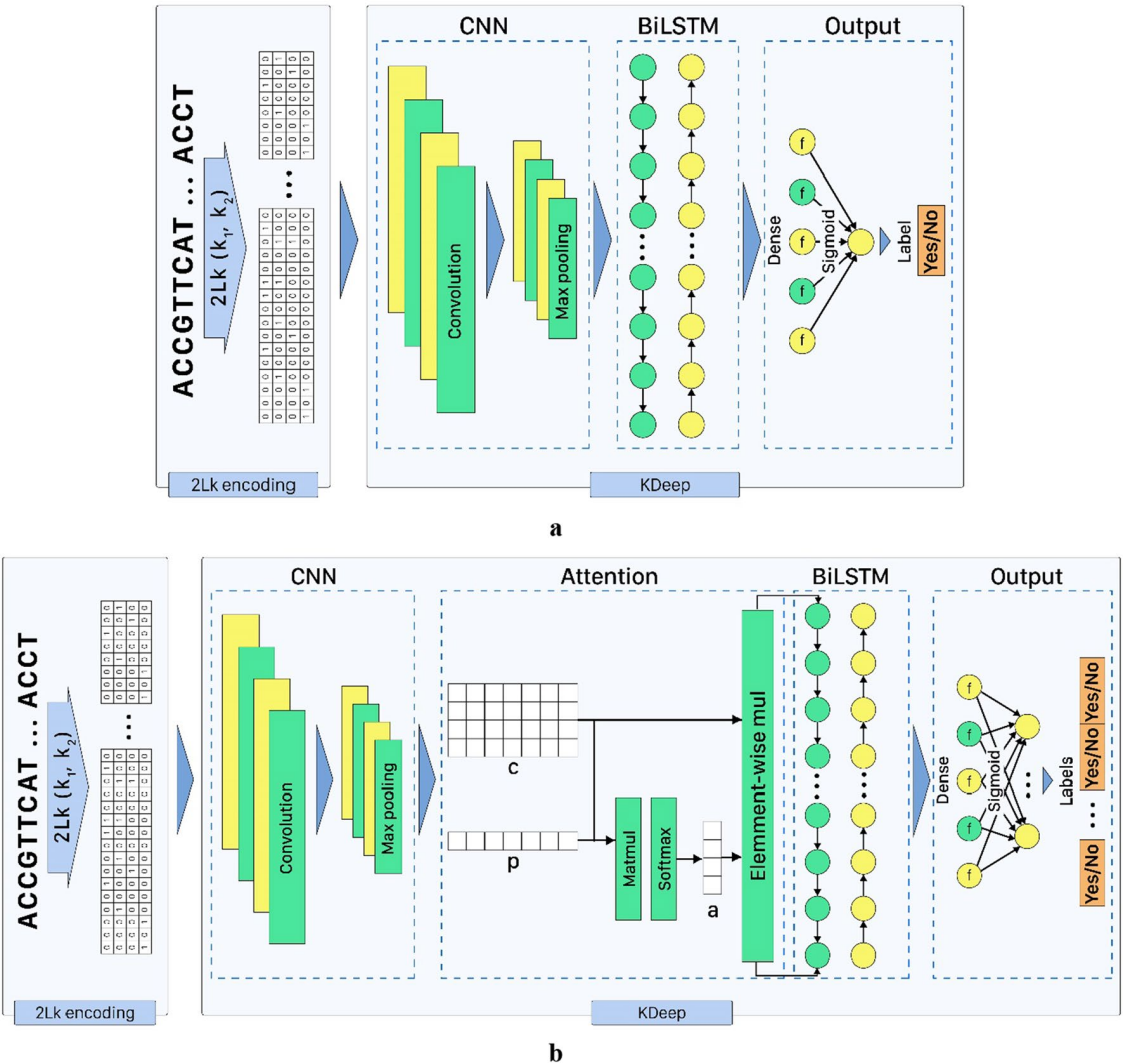
Learning model consists of two main parts that consists CNN and LSTM networks; **a** KDeep, **b** KDeep +

We implement our architecture, called KDeep, using TensorFlow version 2.8. This architecture includes a CNN followed by an LSTM network. At the first step, an encoded DNA/RNA sequence feed the CNN layer responsible for scanning and identifying the input features. In order to prevent the vanishing gradient problem, a Rectified Linear Unit (ReLU) is then employed to sparsify the output of the convolution layer and maintain only the positive matches [40]. Following the convolutional layer, a max pooling operation is performed to reduce the dimensionality and provide invariance to small strand shifts by pooling adjacent positions within a small window. To prevent over fitting, the dropout layer is then used to randomly set input units to zero with a frequency of rate at each step during the training phase [41]. The dependency between the extracted features in the preceding layers is then determined using a BiLSTM layer. Moreover, the issue of vanishing gradients can be resolved by the LSTM, a particular kind of RNN network. In this manner, the long-term interdependency of features can be detected as well. BiLSTM makes use of contextual data from both sides to discover hidden dependencies [24]. In order to avoid over fitting, the dropout layer is applied following the BiLSTM and is used to erratically set input units to zero with a frequency of rate at each step throughout the training period. Two dense layers are utilized for classification at the final step, while the number of neurons in the final dense layer depends on the number of classes. The KDeep learning model is depicted in Fig. 7a.

Moreover, we implement the attention mechanism for DNA learning following the CNN layer of the KDeep architecture. This architectural enhancement improved the performance results, since the attention mechanism can assign different weight scores to each fragment of an input strand to focus on more important fragments when generating outputs. In other words, taking advantages of attention scores, we can specify which part of the input is essential to generate outputs [42]. The learning model is depicted in Fig. 7b, which is called KDeep + , while its parameters are listed in Additional file 3: Table S1.

### Visualization

As a key advantage of the proposed learning model, KDeep and KDeep + can find binding site motifs, since the proposed CNN-based network provides high interpretability, compared to the alternative networks. Specifically, we extract motifs using the first layer of CNN kernels, and the position weight matrix (PWM) is generated using the convolution filters. A convolution filter scans sample strands in all conceivable directions. Since the filter has a size of F with one stride, it can scan an S bps strand at S—F + 1 = P sites and choose the highest value while obtaining p activation values. The maximum value indicates that this sub-strand (motif) plays a significant role in decision-making by the learning model. As a result, the sub-strand associated with that activation is returned to its original nucleotide form through the decoding process. Afterwards, the remaining samples with S bps strands goes through the same process, with the valid sub-strands being aligned to create a position frequency matrix (PFM), which is then transformed to a PWM. In this manner, an output file is generated containing all the extracted motifs. For RNA datasets, we use the TOMTOM tool [43] which includes motifs of RNA strands in its RNA and Jaspar [44] (for DNA) dataset, to compare these patterns against the known motifs.

### Supplementary Information

**Additional file 1:** Performance evaluation of DanQ, KDeep, and KDeep+ models on 919 targets. The area under the receiver operating characteristic curve (auROC) and the area under the precision-recall curve (auPR) values for each target are accessible, demonstrating the performance of the models.

**Additional file 2:** Comparing auROCof five encodings methods: one-hot, word2vec (50), word2vec (100), 2Lk (3, 2), and 2Lk (3, 3) with fixed predictor architecture KDeep.

**Additional file 3: Figure S1.** An example of CGR and FCGR encoding methods. **Table S1.** Details of the KDeep models adopted for DNA and RNA data. **Table S2.** Details of 24 sub-datasets of RBP-24 dataset. **Figure S2.** The hardware performance adopting five encoding methods: one-hot, word2vec (50), word2vec (100), 2Lk (3, 2), and 2Lk (3, 3) with the same architecture for dataset1 of RBP-31; a) GPU Power Usage (W), b) GPU Power Usage (%), c) GPU Memory Allocated (%), d) GPU Time Spent Accessing Memory (%), e) GPU Temp (℃), f) GPU Utilization (%), g) Network Traffic (bytes), h) Disk Utilization (%), i) Process CPU Threads In Use, j) Process Memory Available (non-swap) (MB), k) Process Memory In Use (non-swap) (%), l) Process Memory In Use (non-swap) (MB), m) System Memory Utilization (%), n) CPU Utilization (%). **Figure S3.** The hardware performance adopting five encoding methods: one-hot, word2vec (50), word2vec (100), 2Lk (3, 2), and 2Lk (3, 3) with the same architecture for dataset 1 of RBP-24; a) GPU Power Usage (W), b) GPU Power Usage (%), c) GPU Memory Allocated (%), d) GPU Time Spent Accessing Memory (%), e) GPU Temp (℃), f) GPU Utilization (%), g) Network Traffic (bytes), h) Disk Utilization (%), i) Process CPU Threads In Use, j) Process Memory Available (non-swap) (MB), k) Process Memory In Use (non-swap) (%), l) Process Memory In Use (non-swap) (MB), m) System Memory Utilization (%), n) CPU Utilization (%). **Table S3.** Comparing auROC and auPR of five encodings methods: one-hot, word2vec (50), word2vec (100), 2Lk (3, 2), and 2Lk (3, 3) with fixed predictor architecture KDeep for first dataset of RBP-24. **Table S4.** Hyperparameter and model detail and samples number. **Figure S4** Total number of trainable parameters for 5 sequence encoding method – RNA datasets. **Figure S5.** Comparing auROC and auPR distribution of three binding site predictor methods for DNAsite dataset; a) auROC for Dnase samples of DNAsite dataset, b) auPR for Dnase samples of DNAsite dataset, c) auROC for TF samples of DNAsite dataset, d) auPR for TF samples of DNAsite dataset, e) auROC for Histone samples of DNAsite dataset, f) auPR for Histone samples of DNAsite dataset. **Figure S6.** Total number of trainable parameters for 3 DNA binding site predictor methods. **Figure S7.** Interpretation process in the KDeep method. **Figure S8.** Extracted RNA motifs, as compared to the known motifs, using the TOMTOM tool with KDeep for jaspar dataset; a to e for experience 2 for RBP-24 datasets, f to m for experience 4 of RBP-31 datasets, n to x for experience 20 of RBP-31 datasets. **Figure S9.** Extracted DNA motifs, as compared to the known motifs, using the TOMTOM tool with KDeep for jaspar dataset. **Figure S10.** Extracted DNA motifs, as compared to the known motifs, using the TOMTOM tool with KDeep+ for jaspar dataset. **Figure S11.** Heatmap of output score of attention layer in KDeep+ for DNA. a) Heatmap is related to all the samples that have at least one of the 14 (399, 383, 382, 377, 373, 351, 314, 313, 392, 269, 223, 184) TF type labels positive. b) Heatmap is related to one sample that have at least one of the 14 (399, 383, 382, 377, 373, 351, 314, 313, 292, 269, 223, 184) TF type labels positive and it shows that the middle of this sample has the ability of protein binding.

**Additional file 4:** Performance comparison of KDeep against other RBP-site predictors on the RBP-24 dataset. The evaluation includes DeepRKE-, DeepRKE+, GraphProt, deepnet-rbp, DeepBind, mmCNN, and iDeepV.

**Additional file 5:** Performance comparison of KDeep against other RBP-site predictors on the RBP-31 dataset. The evaluation includes DeepRKE-, DeepRKE+, GraphProt, iDeepV, DeepBind, iONMF, and iDeepS.

## Data Availability

Source code is freely available for download at https://github.com/nazanintbtb/KDeep, implemented in python, and supported on Linux and MS Windows.
