# Supplementary material for: KDeep: a new memory-efficient data extraction method for accurately predicting DNA/RNA transcription factor binding sites
Source: J Transl Med. 2023 Oct 16;21:727. doi: 10.1186/s12967-023-04593-7 (PMC10580661; doi:10.1186/s12967-023-04593-7)
Supplement: Supplementary file 3 — Additional file 3: Figure S1. An example of CGR and FCGR encoding methods. Table S1. Details of the KDeep models adopted for DNA and RNA data. Table S2. Details of 24 sub-datasets of RBP-24 dataset. Figure S2. The hardware performance adopting five encoding methods: one-hot, word2vec (50), word2vec (100), 2Lk (3, 2), and 2Lk (3, 3) with the same architecture for dataset1 of RBP-31; a) GPU Power Usage (W), b) GPU Power Usage (%), c) GPU Memory Allocated (%), d) GPU Time Spent Accessing Memory (%), e) GPU Temp (℃), f) GPU Utilization (%), g) Network Traffic (bytes), h) Disk Utilization (%), i) Process CPU Threads In Use, j) Process Memory Available (non-swap) (MB), k) Process Memory In Use (non-swap) (%), l) Process Memory In Use (non-swap) (MB), m) System Memory Utilization (%), n) CPU Utilization (%). Figure S3. The hardware performance adopting five encoding methods: one-hot, word2vec (50), word2vec (100), 2Lk (3, 2), and 2Lk (3, 3) with the same architecture for dataset 1 of RBP-24; a) GPU Power Usage (W), b) GPU Power Usage (%), c) GPU Memory Allocated (%), d) GPU Time Spent Accessing Memory (%), e) GPU Temp (℃), f) GPU Utilization (%), g) Network Traffic (bytes), h) Disk Utilization (%), i) Process CPU Threads In Use, j) Process Memory Available (non-swap) (MB), k) Process Memory In Use (non-swap) (%), l) Process Memory In Use (non-swap) (MB), m) System Memory Utilization (%), n) CPU Utilization (%). Table S3. Comparing auROC and auPR of five encodings methods: one-hot, word2vec (50), word2vec (100), 2Lk (3, 2), and 2Lk (3, 3) with fixed predictor architecture KDeep for first dataset of RBP-24. Table S4. Hyperparameter and model detail and samples number. Figure S4 Total number of trainable parameters for 5 sequence encoding method – RNA datasets. Figure S5. Comparing auROC and auPR distribution of three binding site predictor methods for DNAsite dataset; a) auROC for Dnase samples of DNAsite dataset, b) auPR for Dnase samples of DNAsite dataset, c) auROC for TF [file 12967_2023_4593_MOESM3_ESM.docx]

**KDeep: a new memory-efficient data extraction method for accurately predicting DNA/RNA transcription factor binding sites**

Saeedeh Akbari Rokn Abadi^1, b^, SeyedehFatemeh Tabatabaei^1, b^, Somayyeh Koohi^1, *^

^1^ Department of Computer Engineering, Sharif University of Technology, Tehran, Iran

^b^ These authors contributed equally

^*^ Correspondence: E-mail: [koohi@sharif.edu](mailto:koohi@sharif.edu)

Contents

[FCGR algorithm 2](#_Toc126778592)

[Details of predictor model architecture 3](#_Toc126778593)

[Datasets 4](#_Toc126778594)

[Hardware resources 6](#_Toc126778595)

[Investigating the impact of sequence encoding method on the predictor performance 10](#_Toc126778596)

[KDeep’s performance for DNA datasets 11](#_Toc126778597)

[Visualization 13](#_Toc126778598)

[KDeep result for RNA dataset 14](#_Toc126778599)

[KDeep result for DNA dataset 17](#_Toc126778600)

[KDeep+ result for DNA dataset 18](#_Toc126778601)

# FCGR algorithm

In many classification studies of genomic strands, an alignment-free encoding method called Chaos Game Representation (CGR) is used to represent the strands. CGR is an iterative mapping technique based on Markov probability table that processes strands of units, such as nucleotides in a DNA/RNA strand or amino acids in a protein, in order to find the coordinates of their position in a continuous space. CGR space produced by a strand, with $n$ unique alphabets, is a polygon bounded by *n* possible alphabets. Specifically, for the genomic strands, CGR space is a plane bounded by four possible nucleotides (i.e. Adenine (A), Thymine (T) in DNA or Uracil (U) in RNA, Cytosine (C), and Guanine (G)) as vertices of a binary square. Rather than arranging nucleotides in a linear way, CGR, as a novel holistic approach, provides a two-dimensional visual representation of a DNA/RNA strands. Specifically, the CGR position $CGR_{i}$ of each alphabet $s_{i}$ of a strands $s$ of length $l_{S}$ is calculated by moving a pointer to half the distance between the previous position and the current binary representation (Eq. S 1). For DNA/RNA strands, the binary square CGR vertices are assigned to the four nucleotides as A = lower left (0, 0), T|U= lower right (0, 1), G = upper right (1, 1), and C = upper left (1, 0). The procedure is illustrated in Fig. S 1.

| ${CGR}_{i}=0.5 . \left( {CGR}_{i-1}+P \right)$  $\mathrm{with} i=1,\ldots, \left\vert s \right\vert and {CGR}_{0}=\left( 0.5,0.5 \right) and P\in\{A,C,G,T\vert U\}$ | Eq. S 1 |
| --- | --- |

CGR extracts important information from the number of k-mers. Each k-mer defines a coordinate space in a $4^{k}$ dimensions vector space, assuming k-mers of length $k$, and four possible values for each spot (A, C, G, T|U). As proven, the abundance of all k-mers (oligonucleotides of length k) in a given strands can be determined by dividing the CGR space with a grid of appropriate size, and then, counting the k-mer’s occurrences in each quadrant. In order to obtain the frequency matrix of oligonucleotides of length $k$, a $2^{k}\times2^{k}$grid must be used and this largely covers all $4^{k}$ possible k-mers. This matrix is called FCGR (Frequency of CGR). **Error! Reference source not found.** depicts an example of FCGR generation from a sample strand.


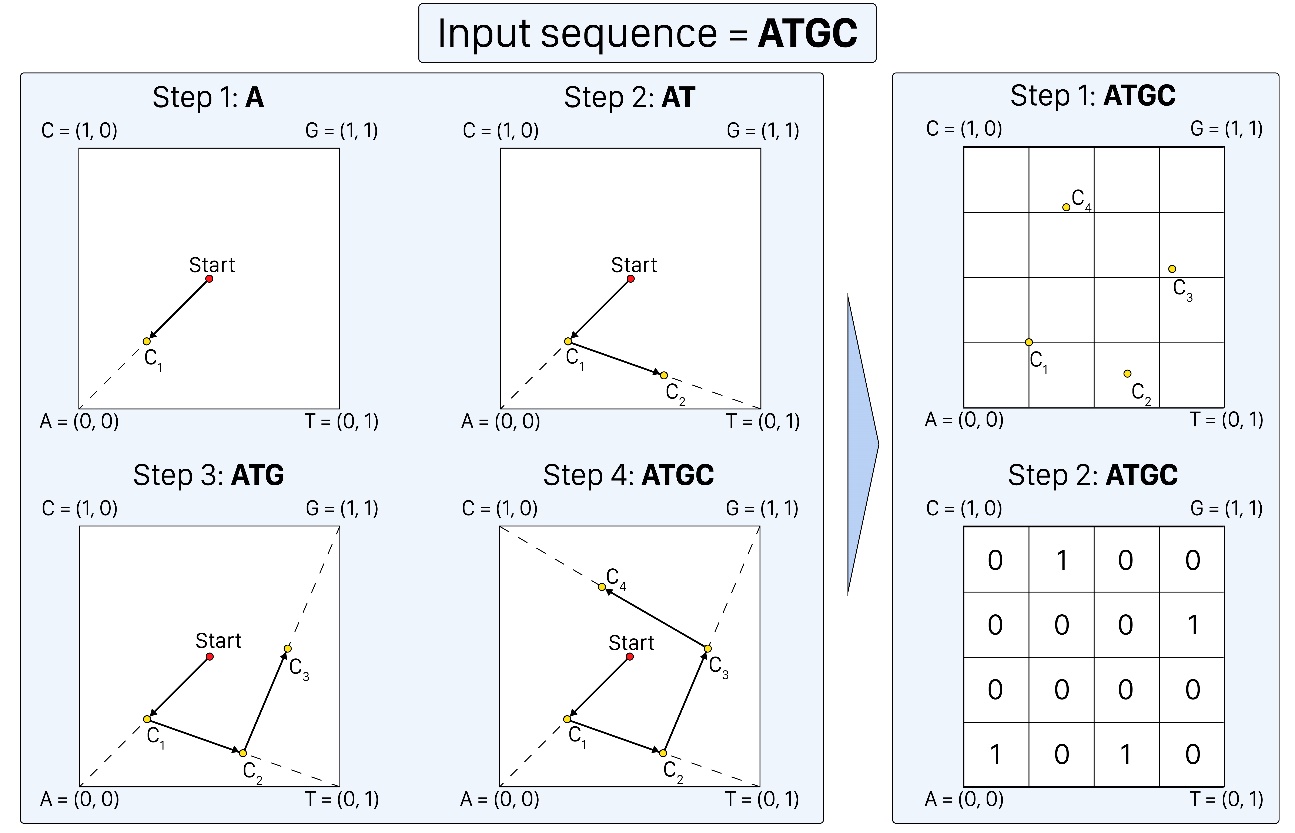


Fig. S 1 An example of CGR and FCGR encoding methods

# Details of predictor model architecture

Table S 1 Details of the KDeep models adopted for DNA and RNA data

| Hyper-parameters | KDeep | | KDeep, KDeep+ |
| --- | --- | --- | --- |
| Sequence type | RNA | | DNA |
| K_mer size | k_1_=3 , k_2_=2 (2Lk_3,2_) | k_1_=3 , k_2_=3 (2Lk_3,3_) | k_1_=3 , k_2_=3 (2Lk_3,3_) |
| Convolution filter size | 64 | | 320 |
| Convolution kernel size | 12 | | 26 |
| Pooling size | 2 | | 13 |
| Stride | 1 | | 13 |
| Dropout factor | 0.2 | | 0.2 |
| LSTM unit size | 32 | | 320 |
| Dropout factor | 0.5 | | 0.5 |
| Dense unit size | 925 | | 925 |
| Dense unit size | 1 | | 919 |
| Batch-size | 100,300 | | 100 |
| Epoch | 60 | | 60 |

# Datasets

In order to evaluate various binding scenarios, three significant benchmark datasets are used. It should be noted that KDeep is built to predict the protein binding sites to both DNA and RNA strand types. For the purpose of testing the KDeep method's ability to predict RNA binding sites, the binary-label classification datasets RBP-24 and RBP-31 and to predict DNA binding sites, the multi-label classification dataset DNAsite are selected.

The dataset related to the DNA sequence section is the same dataset used by the famous DanQ method, which was introduced by the DeepSea method. These models were trained on 521,636,200 bp of sequences (17% of the genome) with at least one TF binding event. Each training sample consisted of a 1,000-bp sequence centered on a 200-bp bin and paired with a label vector for 919 chromatin features. Training and testing sets were split by chromosomes, with chromosomes 8 and 9 excluded from the training set to test chromatin feature prediction performance. The full list of all chromatin profile used files is provided in Supplementary Table 1 of [1].

RBP-31 was originally obtained from DoRiNA [2] and iCount,( http://icount.biolab.si/) and was later made available on GitHub by the developers of iONMF [3]. The positive sites in the dataset represent nucleotides that were identified as being within clusters of interaction sites derived from CLIP-seq, while the negative sites were extracted from genes not participating in the protein-RNA interaction process in any of the 31 experiments. Each experiment in the dataset consists of 40,000 examples divided into 30,000 examples for training and 10,000 for testing. The dataset was used to evaluate the ability of different architectures to identify RNA-binding sites [4].

The RBP-24 dataset used in the paper is a benchmark dataset for predicting RBP binding sites. The dataset consists of 24 sets of HITS-CLIP, PAR-CLIP, and iCLIP-derived binding sites, which were curated by doRiNA and an additional set of PTB HITS-CLIP binding sites was taken from a previous study. The binding sites were derived from various RBPs, including Ago1-4, IGF2BP1-3, ELAVL1, ALKBH5, C17ORF85, C22ORF28, CAPRIN1, EWSR1, FUS, HNRNPC, MOV10, PTB, PUM2, QKI, SFRS1, TAF15, TDP-43, TIA1, TIAL1, and ZC3H7B [5]. The numbers of train samples and positive and negative samples for the 24 RBP-24 sub-datasets, which have the various number of train samples, are displayed in Table S 1.

Table S 2 Details of 24 sub-datasets of RBP-24 dataset

| RBP-24 | # Positive samples | # Negative samples | #Train samples |
| --- | --- | --- | --- |
| ALKBH5 PAR-CLIP | 1213 | 1197 | 2410 |
| C17ORF85 PAR-CLIP | 1860 | 1849 | 3709 |
| C22ORF28 PAR-CLIP | 9369 | 9136 | 18505 |
| CAPRIN1 PAR-CLIP | 8140 | 7901 | 16041 |
| Ago2 HITS-CLIP | 48,095 | 44,251 | 92346 |
| ELAVL1 HITS-CLIP | 8595 | 8436 | 17031 |
| SFRS1 HITS-CLIP | 19,438 | 17,195 | 36633 |
| HNRNPC iCLIP | 21,472 | 19,794 | 41266 |
| TDP43 iCLIP | 92,031 | 75,079 | 167110 |
| TIA1 iCLIP | 18,049 | 16,135 | 34184 |
| TIAL1 iCLIP | 42,332 | 36,652 | 78984 |
| Ago1-4 PAR-CLIP | 36,902 | 31,310 | 68212 |
| ELAVL1 PAR-CLIP(B) | 9464 | 9283 | 18747 |
| ELAVL1 PAR-CLIP (A) | 27,275 | 23,974 | 51249 |
| EWSR1 PAR-CLIP | 16,292 | 14,720 | 31012 |
| FUS PAR-CLIP | 34,581 | 31,480 | 66061 |
| ELAVL1 PAR-CLIP(C) | 125,202 | 113,686 | 238888 |
| IGF2BP1-3 PAR-CLIP | 8539 | 6838 | 15377 |
| MOV10 PAR-CLIP | 13,793 | 12,987 | 26780 |
| PUM2 PAR-CLIP | 9116 | 8227 | 17343 |
| QKI PAR-CLIP | 10,276 | 9142 | 19418 |
| TAF15 PAR-CLIP | 7298 | 6606 | 13904 |
| PTB HITS-CLIP | 44,574 | 43,700 | 88274 |
| ZC3H7B PAR-CLIP | 20,962 | 20,018 | 40980 |

# Hardware resources

| 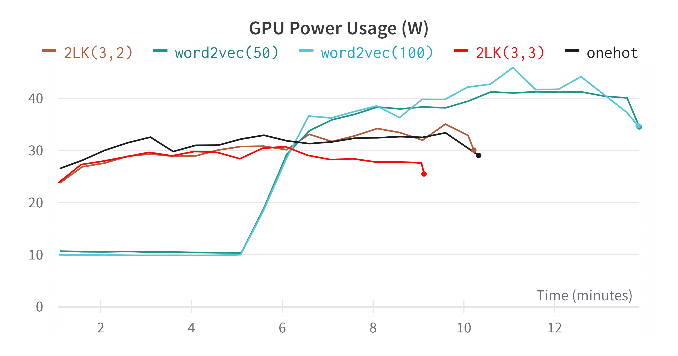 | 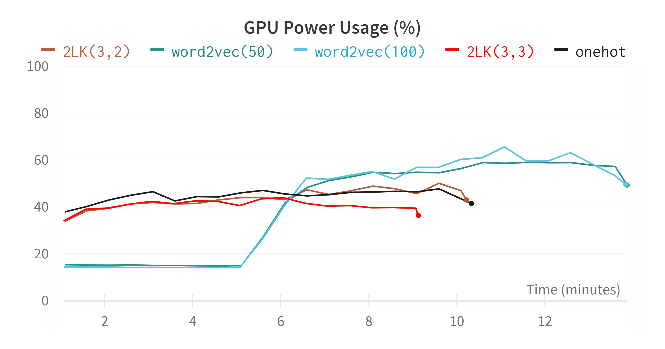 |
| --- | --- |
| **a** | **b** |
| 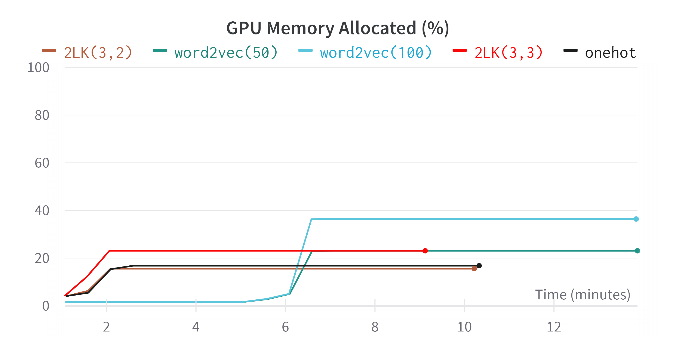 | **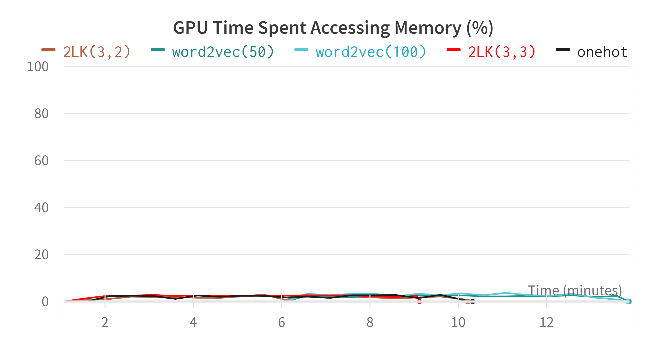** |
| **c** | **d** |
| **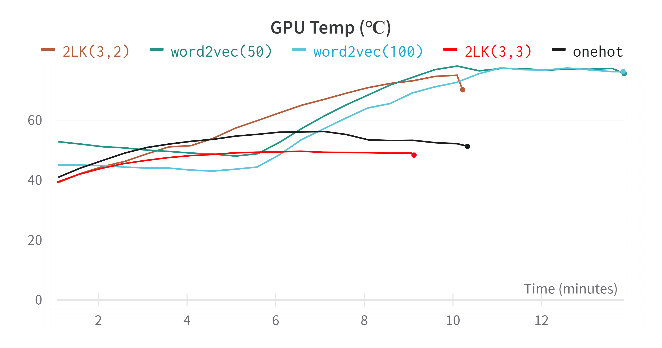** | **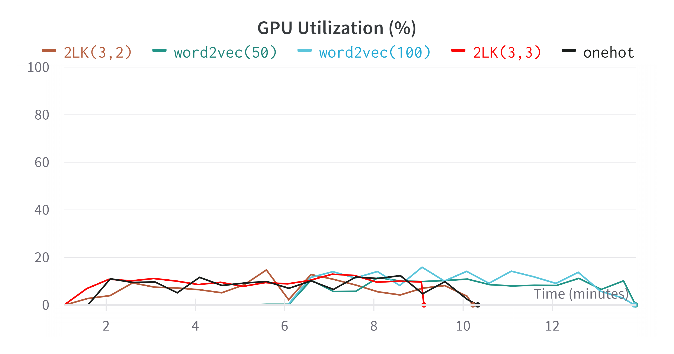** |
| **e** | **f** |
| **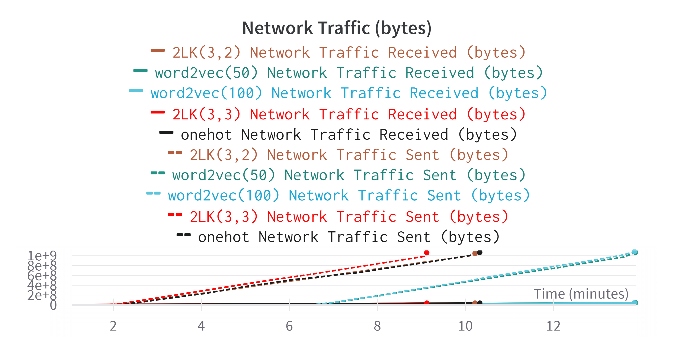** | **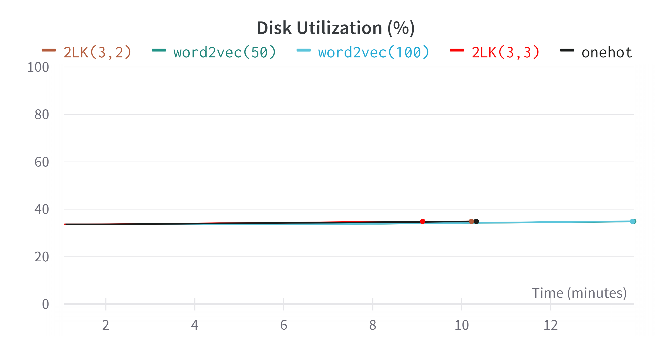** |
| **g** | **h** |
| **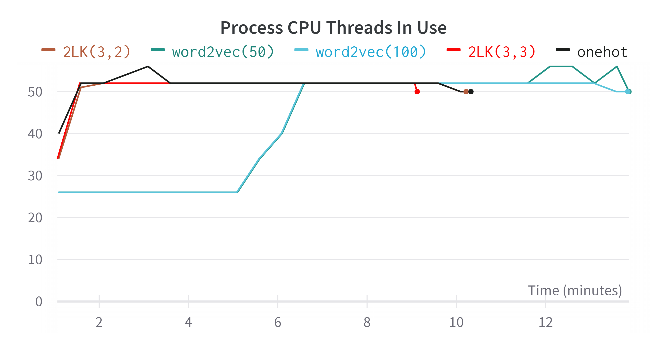** | **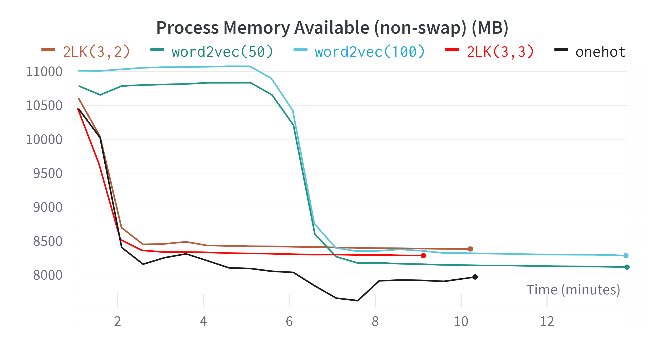** |
| **i** | **j** |
| **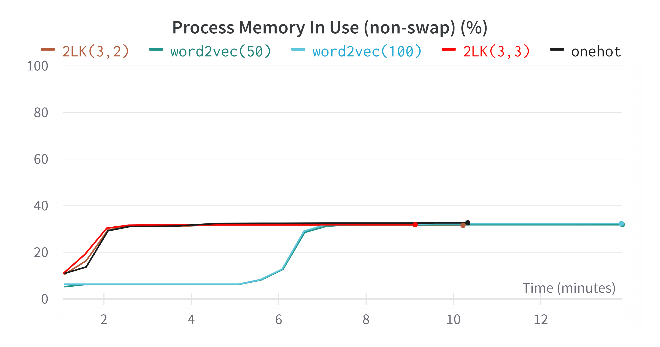** | **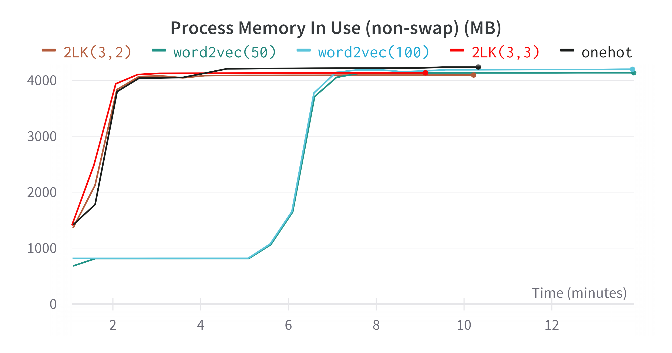** |
| **k** | **l** |
| **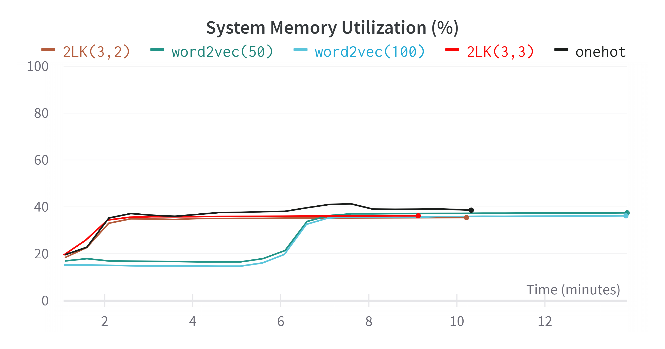** | **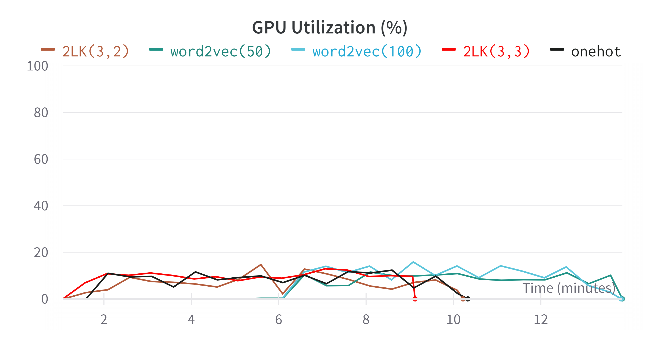** |
| **m** | **n** |

Fig. S 2 The hardware performance adopting five encoding methods: one-hot, word2vec (50), word2vec (100), 2Lk (3, 2), and 2Lk (3, 3) with the same architecture for dataset1 of RBP-31; a) GPU Power Usage (W), b) GPU Power Usage (%), c) GPU Memory Allocated (%), d) GPU Time Spent Accessing Memory (%), e) GPU Temp (℃), f) GPU Utilization (%), g) Network Traffic (bytes), h) Disk Utilization (%), i) Process CPU Threads In Use, j) Process Memory Available (non-swap) (MB), k) Process Memory In Use (non-swap) (%), l) Process Memory In Use (non-swap) (MB), m) System Memory Utilization (%), n) CPU Utilization (%)

| 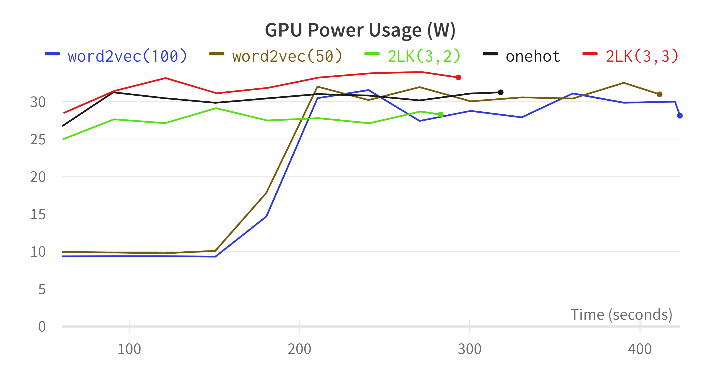 | 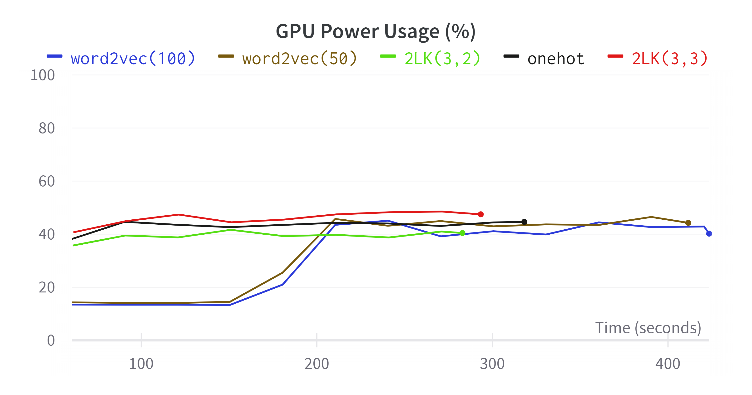 |
| --- | --- |
| **a** | **b** |
| 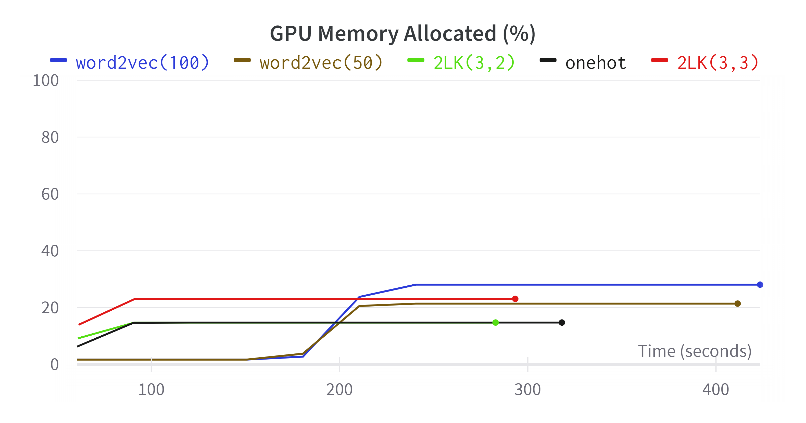 | **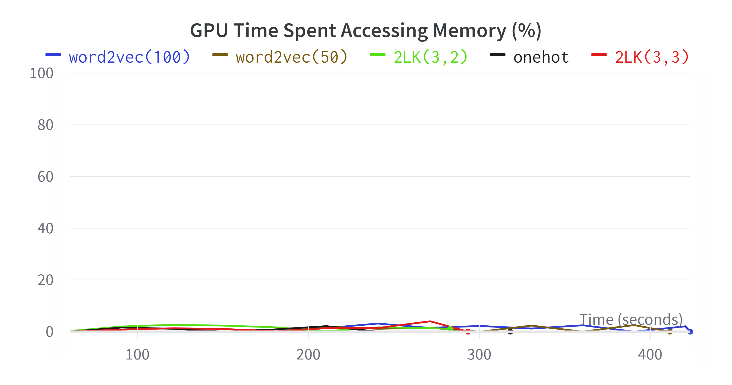** |
| **c** | **d** |
| **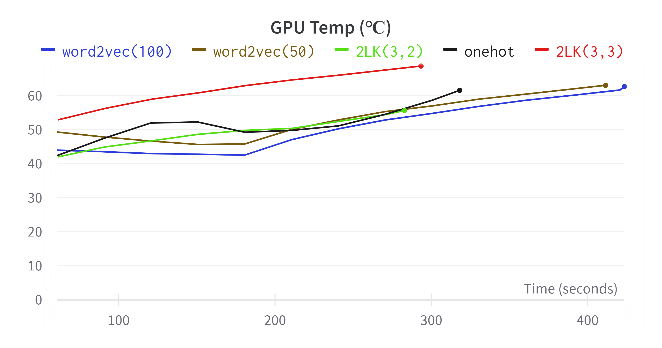** | **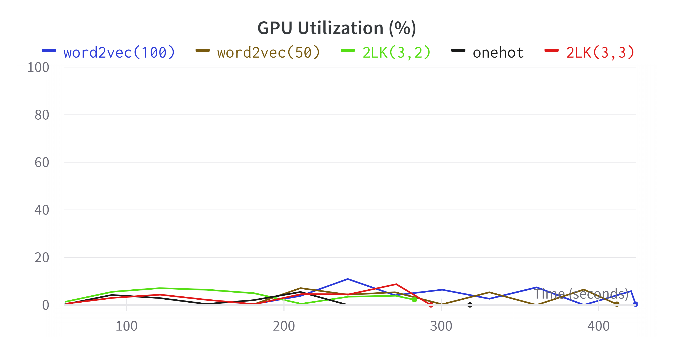** |
| **e** | **f** |
| **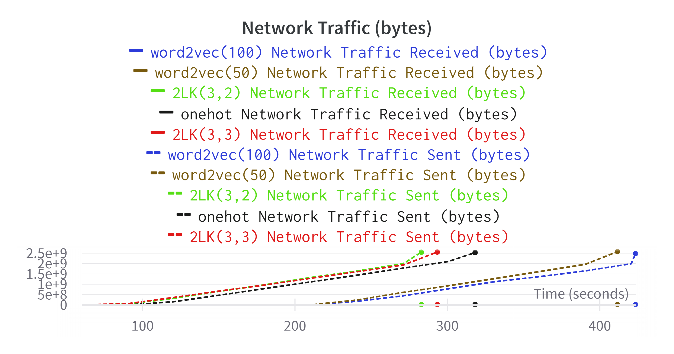** | **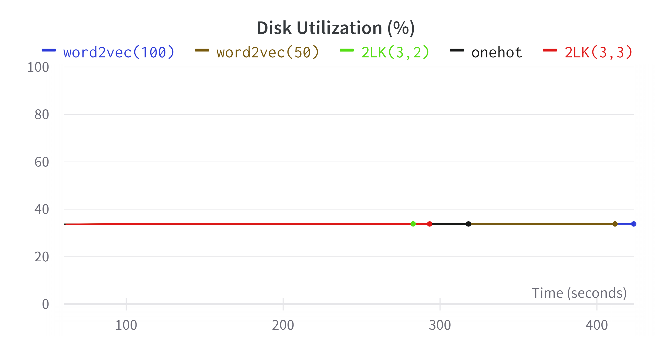** |
| **g** | **h** |
| **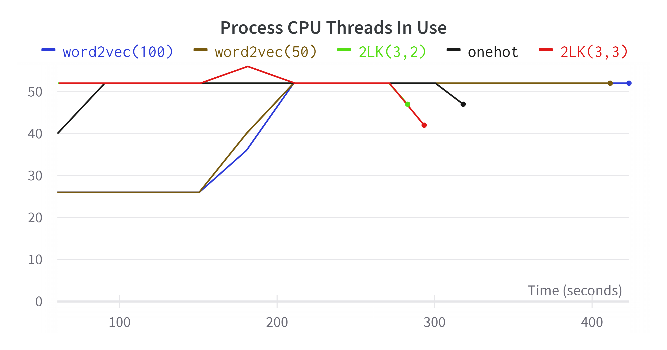** | **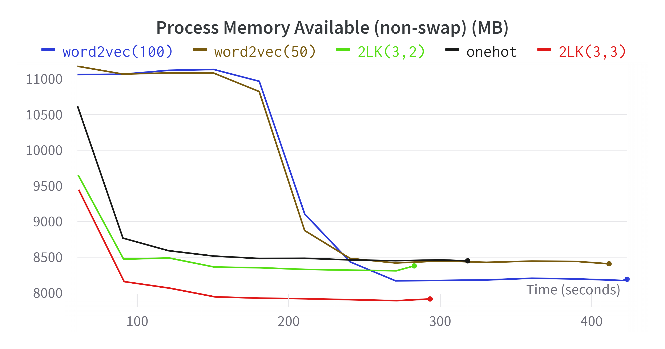** |
| **i** | **j** |
| **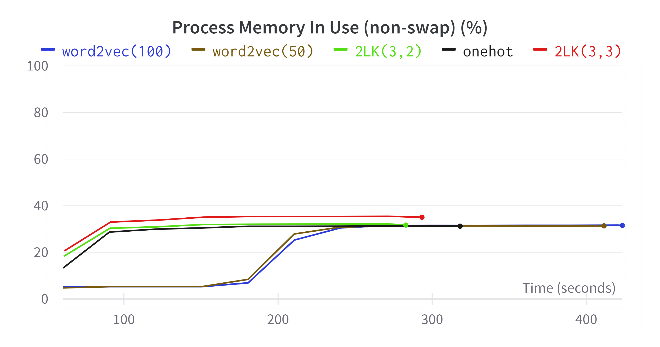** | **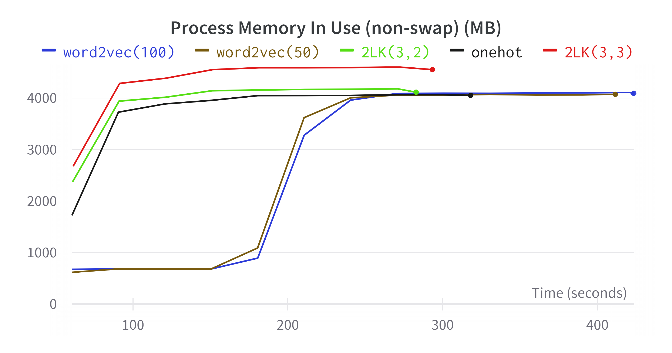** |
| **k** | **l** |
| **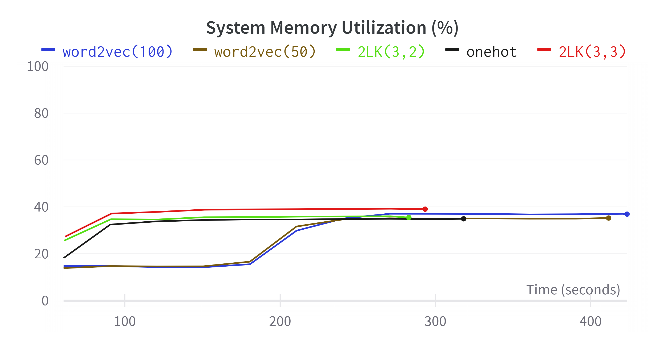** | **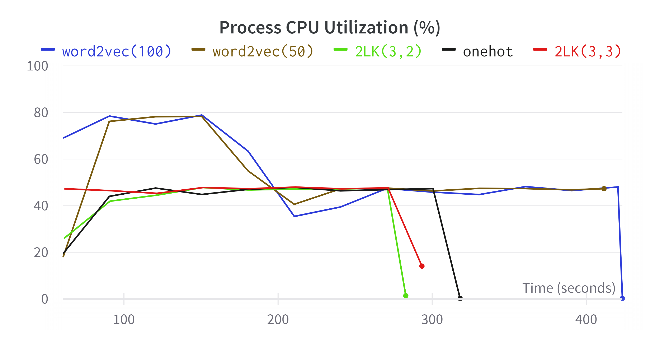** |
| **m** | **n** |

*Fig. S 3 The hardware performance adopting five encoding methods: one-hot, word2vec (50), word2vec (100), 2Lk (3, 2), and 2Lk (3, 3) with the same architecture for dataset 1 of RBP-24; a) GPU Power Usage (W), b) GPU Power Usage (%), c) GPU Memory Allocated (%), d) GPU Time Spent Accessing Memory (%), e) GPU Temp (℃), f) GPU Utilization (%), g) Network Traffic (bytes), h) Disk Utilization (%), i) Process CPU Threads In Use, j) Process Memory Available (non-swap) (MB), k) Process Memory In Use (non-swap) (%), l) Process Memory In Use (non-swap) (MB), m) System Memory Utilization (%), n) CPU Utilization (%)*

Table S 3 Comparing auROC and auPR of five encodings methods: one-hot, word2vec (50), word2vec (100), 2Lk (3, 2), and 2Lk (3, 3) with fixed predictor architecture KDeep for first dataset of RBP-24

| RBP-24 dataset 1 | | | | | | | | | |
| --- | --- | --- | --- | --- | --- | --- | --- | --- | --- |
| 2Lk (3, 3) | | 2Lk (3, 2) | | Word2vec (50) | | Word2vec (100) | | One hot | |
| auROC | auPR | auROC | auPR | auROC | auPR | auROC | auPR | auROC | auPR |
| 0.756 | 0.757 | 0.753 | 0.746 | 0.744 | 0.736 | 0.753 | 0.75 | 0.743 | 0.722 |

Table S 4 Hyperparameter and model detail and samples number

| Model | One CNN layer + One BiLSTM layer |
| --- | --- |
| Filter number | 64 |
| Kernel size | 12 |
| Pooling size and stride size | 2,2 |
| LSTM Unit | 32 |
| 2 dropout layers | 0.2, 0.5 |
| Dense layer | 925 |
| Dens layer | 1 |
| # Epoch | 30 |
| Batch size | 32 |
| #Train, #Test | 2410, 266 |

# Investigating the impact of sequence encoding method on the predictor performance

Fig. S 4 Total number of trainable parameters for 5 sequence encoding method – RNA datasets

# KDeep’s performance for DNA datasets

| 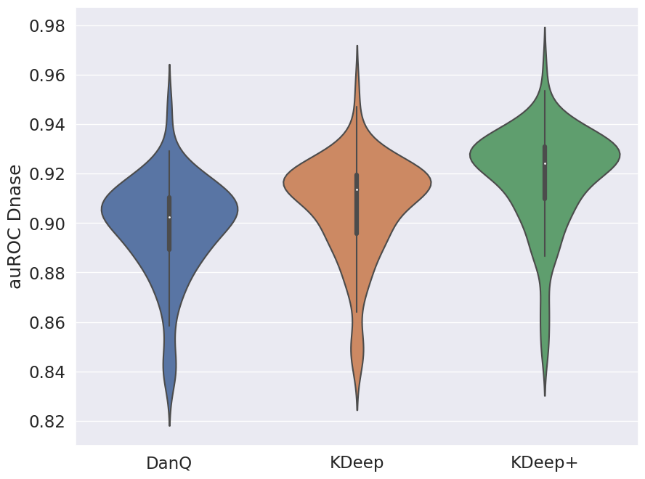 | 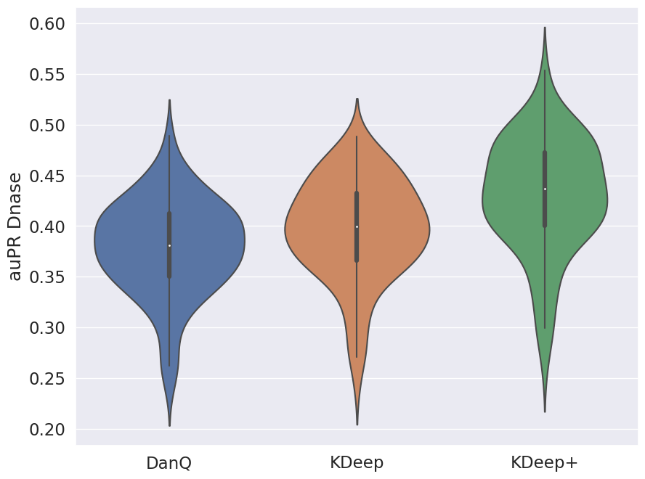 |
| --- | --- |
| \| Method \| Q1 \| Median \| Q3 \| Average \| \| --- \| --- \| --- \| --- \| --- \| \| DanQ \| 0.8890 \| 0.9023 \| 0.9107 \| 0.8982 \| \| KDeep \| 0.8956 \| 0.9134 \| 0.9194 \| 0.9065 \| \| KDeep+ \| 0.9100 \| 0.9241 \| 0.9309 \| 0.9183 \| | \| Method \| Q1 \| Median \| Q3 \| Average \| \| --- \| --- \| --- \| --- \| --- \| \| DanQ \| 0.3497 \| 0.3810 \| 0.4124 \| 0.3789 \| \| KDeep \| 0.3657 \| 0.3994 \| 0.4326 \| 0.3960 \| \| KDeep+ \| 0.4006 \| 0.4367 \| 0.4728 \| 0.4296 \| |
| **a** | **B** |
| 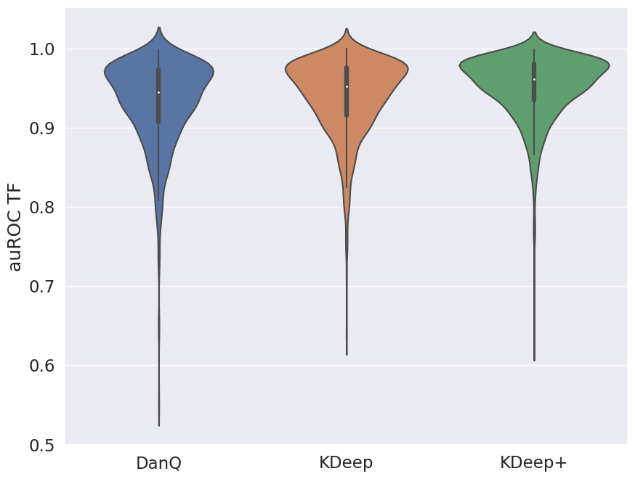 | 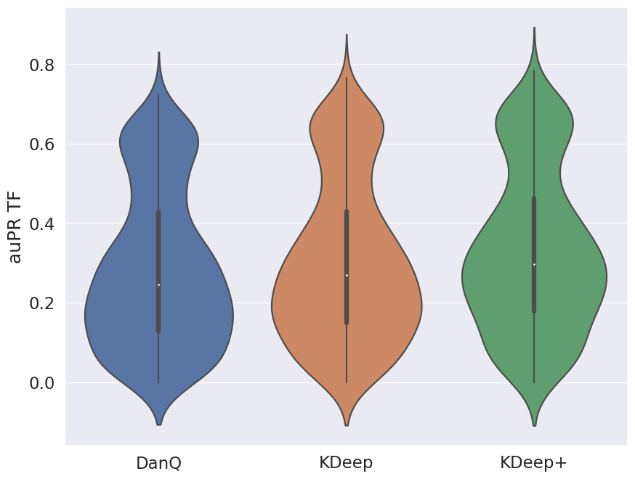 |
| \| Method \| Q1 \| Median \| Q3 \| Average \| \| --- \| --- \| --- \| --- \| --- \| \| DanQ \| 0.9065 \| 0.9445 \| 0.9732 \| 0.9316 \| \| KDeep \| 0.9154 \| 0.9519 \| 0.9764 \| 0.9395 \| \| KDeep+ \| 0.9345 \| 0.9614 \| 0.9810 \| 0.9509 \| | \| Method \| Q1 \| Median \| Q3 \| Average \| \| --- \| --- \| --- \| --- \| --- \| \| DanQ \| 0.1277 \| 0.2453 \| 0.4278 \| 0.2850 \| \| KDeep \| 0.1492 \| 0.2698 \| 0.4309 \| 0.3038 \| \| KDeep+ \| 0.1774 \| 0.2966 \| 0.4616 \| 0.3297 \| |
| **c** | **d** |
|  |  |
|  |  |
|  |  |
|  |  |
|  |  |
| 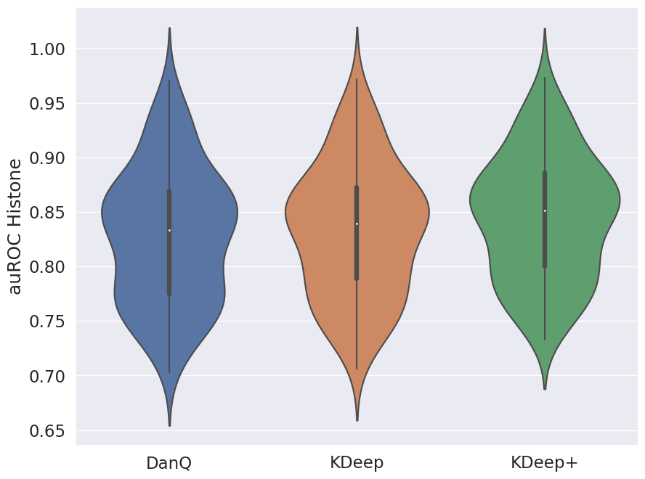 | 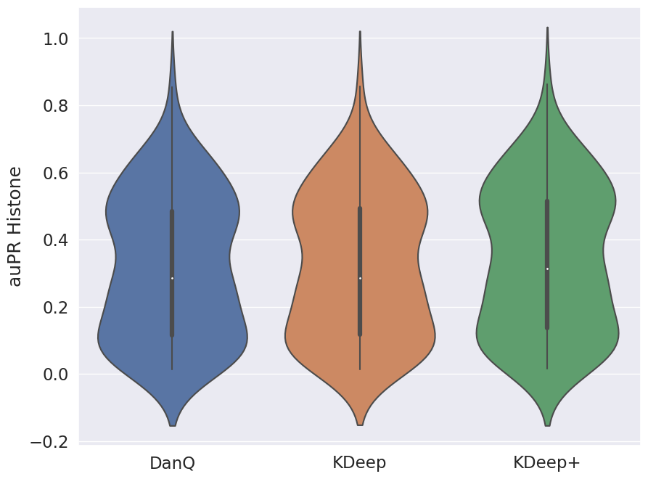 |
| \| Method \| Q1 \| Median \| Q3 \| Average \| \| --- \| --- \| --- \| --- \| --- \| \| DanQ \| 0.7762 \| 0.8352 \| 0.8692 \| 0.8288 \| \| KDeep \| 0.7902 \| 0.8400 \| 0.8732 \| 0.8334 \| \| KDeep+ \| 0.8015 \| 0.8519 \| 0.8862 \| 0.8454 \| | \| Method \| Q1 \| Median \| Q3 \| Average \| \| --- \| --- \| --- \| --- \| --- \| \| DanQ \| 0.1177 \| 0.2890 \| 0.4874 \| 0.3215 \| \| KDeep \| 0.1183 \| 0.2862 \| 0.4936 \| 0.3232 \| \| KDeep+ \| 0.1404 \| 0.3158 \| 0.5148 \| 0.3433 \| |
| **e** | **f** |

Fig. S 5 Comparing auROC and auPR distribution of three binding site predictor methods for DNAsite dataset; a) auROC for Dnase samples of DNAsite dataset, b) auPR for Dnase samples of DNAsite dataset, c) auROC for TF samples of DNAsite dataset, d) auPR for TF samples of DNAsite dataset, e) auROC for Histone samples of DNAsite dataset, f) auPR for Histone samples of DNAsite dataset

Fig. S 6 Total number of trainable parameters for 3 DNA binding site predictor methods

# Visualization

## Interpretation method

The first layer of our learning model consists of a one-dimensional convolutional layer, which is configured with 320 filters for the DNA dataset and 64 filters for the RNA dataset. The size of each filter is determined by the kernel size, stride size, and the length of the input data to this layer. The filters in this layer are responsible for extracting features from the data and assigning weights to each part of the data based on its importance in decision making. Therefore, the effective parts of the input have a higher weight. These features are extracted from the filters, which are known as motifs. The size of each filter can be calculated using Eq. S 1,

| $f_{i}=\frac{B-K}{S}+1$ | Eq. S 1 |
| --- | --- |

where, f represents the number of filters in the first layer, K represents the kernel size, S represents the stride size, and B represents the input data size.

Given that we used the K-mer method to extract words of length 3 in the encoding section and performed 2LK encoding for each word, the length of the input channel for the convolution layer is determined by the 2LK encoding, while the length of the input data, determined by parameter B, is determined by the K-mer method. As a result, the input received by the convolution layer has dimensions with a size of (Batch-size, B, Channel = 2LK-size).

To ensure interpretability, it is important to identify which parts of the filters correspond to which parts of the input data and use the relevant sections to create the PWM (Position Weight Matrix). The PWM is a mathematical model that represents the consensus sequence of a DNA or RNA motif, and is commonly used to identify potential binding sites for transcription factors or other DNA or RNA binding proteins. To create the PWM, we first train the model and then separate the positive test samples, feeding them to the model to extract the filters containing weights. We then scan the positive test samples with a window of length K of the convolution layer kernel and a stride of S, matching them with the corresponding filter weight values. If the number of weights is above 0, it indicates that this scanned part of the samples was important in the decision-making process. We then recover the word's nucleotide using the reverse encoding operation and form the PWM matrix for each filter. Note that in the preprocessing section, we stored the encoding for each word in a dictionary to simplify the reverse encoding and word retrieval operations for interpretability. The fixed coding values for each word also made the interpretability process easier. Fig. S 7 shows this processing details. We now have a PWM matrix for each filter, each pointing to a specific motif. To validate the extracted motifs, we use the TomTom tool, which compares the motifs with well-known databases such as Jaspar and RNA, and measures the degree of alignment of each motif with the motifs in these databases using the E-Value metric. The E-value is a statistical measure that represents the expected number of motifs that would match by chance alone, given the size of the motif database being searched and the length of the query motif. A lower E-value indicates a higher degree of similarity between the query motif and the motifs in the database, and therefore a higher confidence in the match. In other words, a lower E-value suggests that the match is more significant and less likely to have occurred by chance.

As a result, considering the improved prediction accuracy of our method in KDeep and KDeep+, the convolutional layer filters were able to extract features better based on the 2LK encoding property. Therefore, the number of extracted motifs with a lower E-value threshold increased compared to the DanQ method.


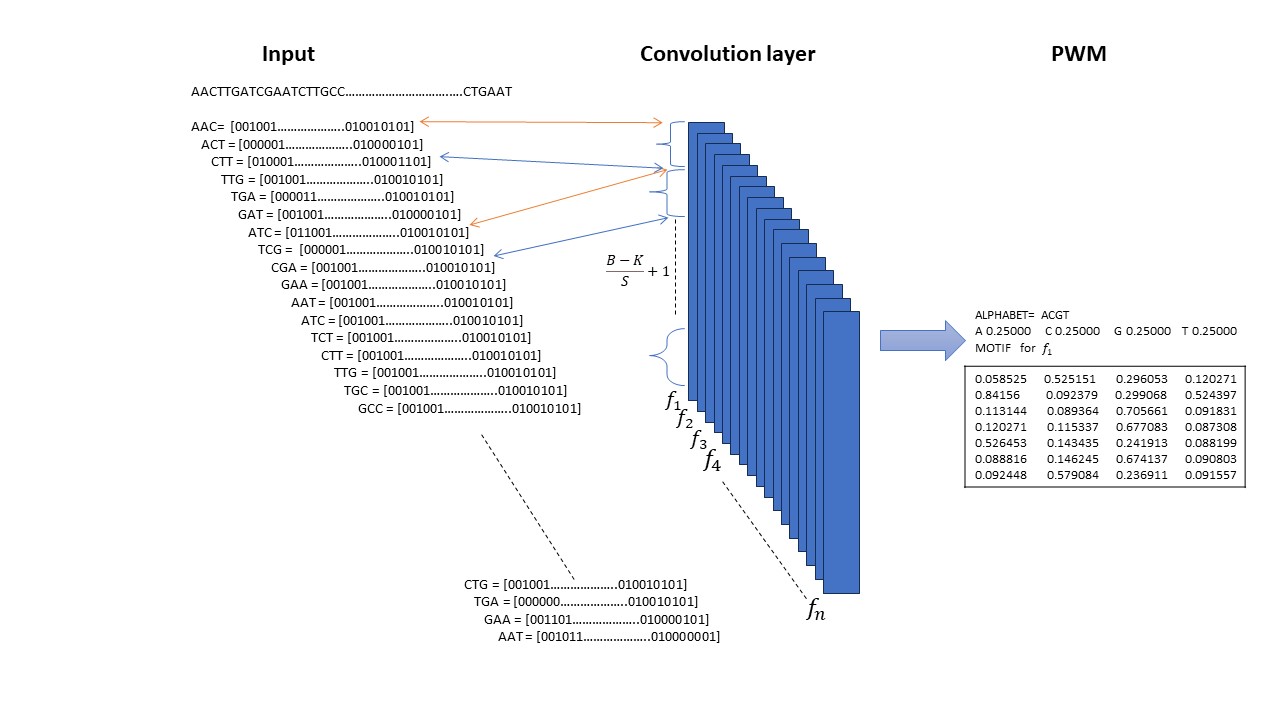


Fig. S 7 Interpretation process in the KDeep method

## KDeep result for RNA dataset

Some of patterns found by KDeep with TomTom evaluation are shown in Fig. S 8. All patterns found for experience 2 of RBP-24, experience 4 of RBP-31, and experience 20 of RBP-31 are also in the KDeep_SM.zip file.

| 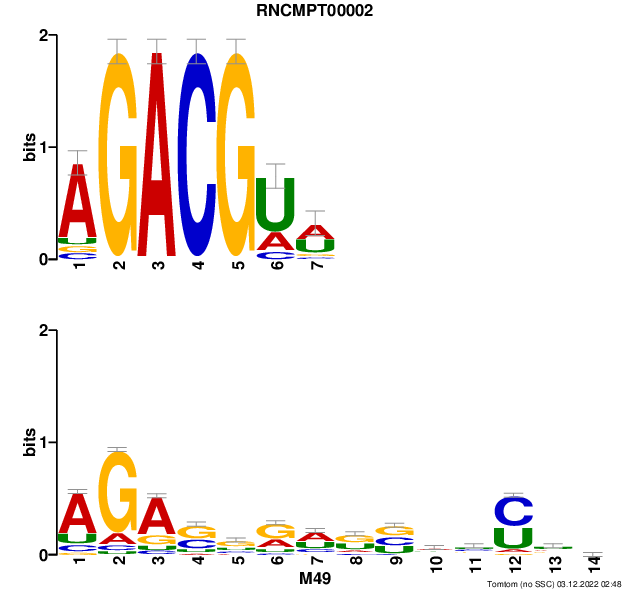 | 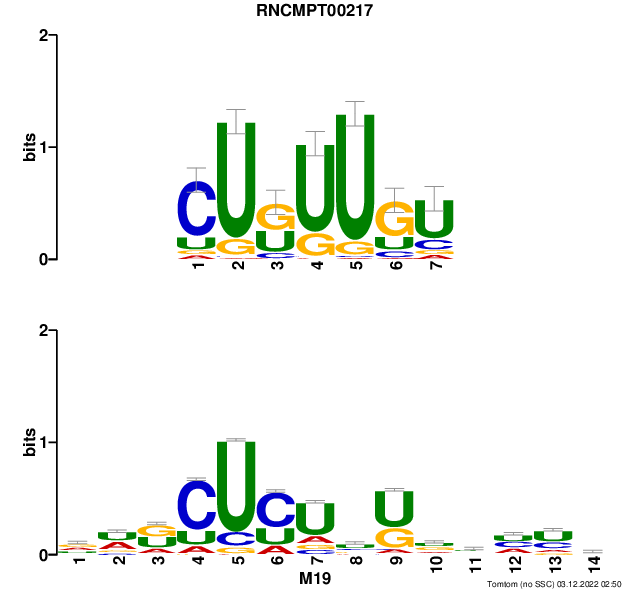 |
| --- | --- |
| a | b |
| 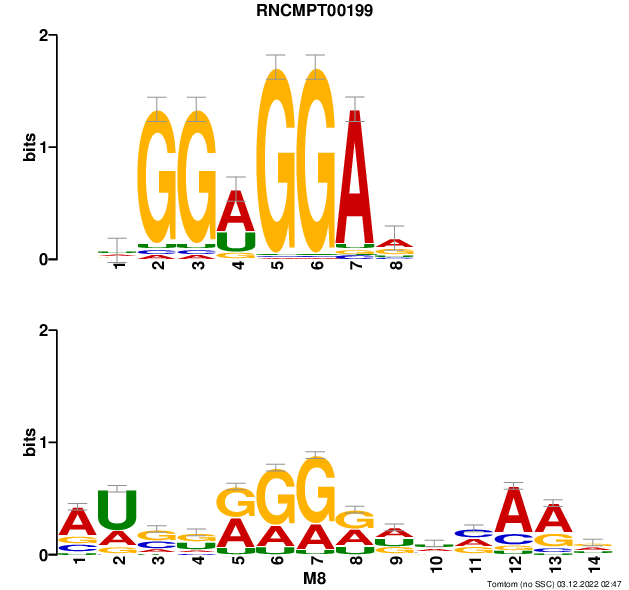 | 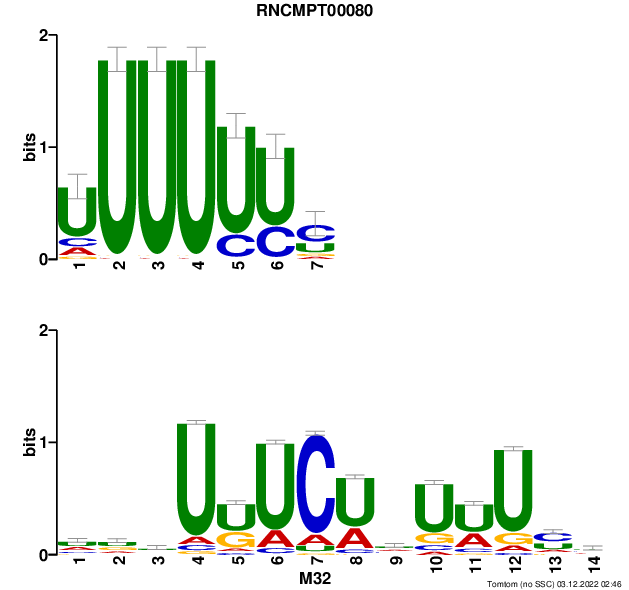 |
| c | d |
| 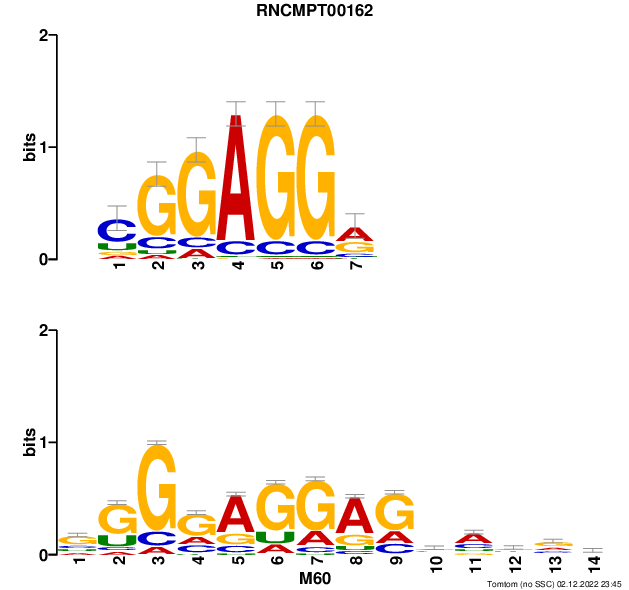 | 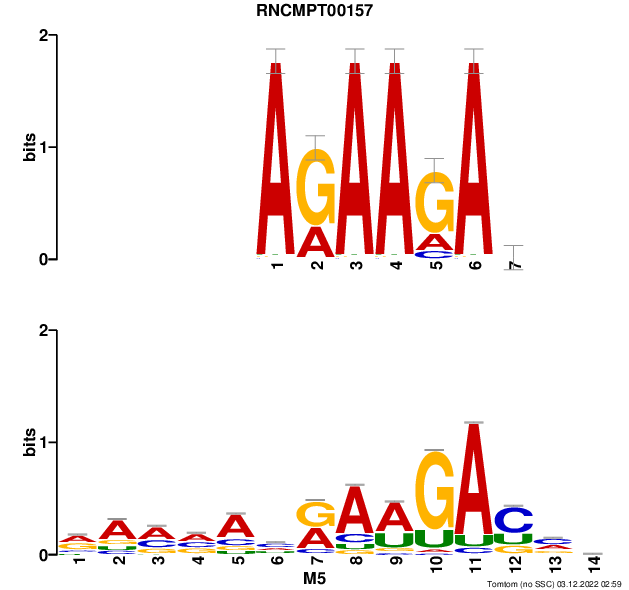 |
| e | f |
| 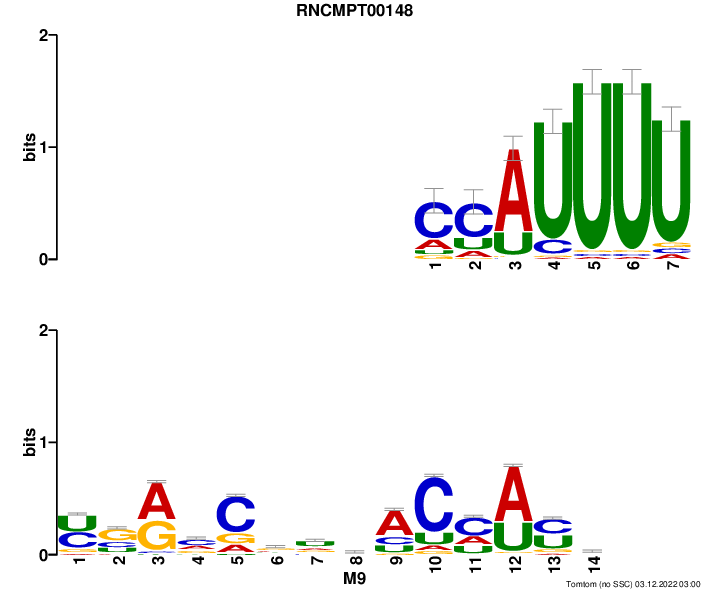 | 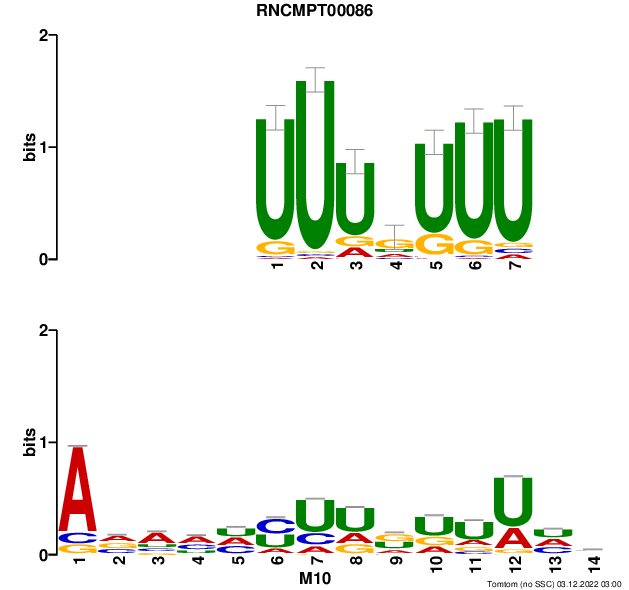 |
| g | h |
| 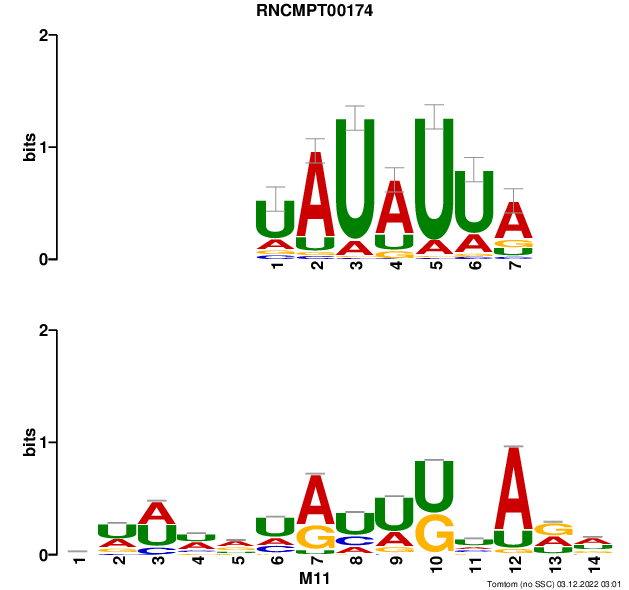 | 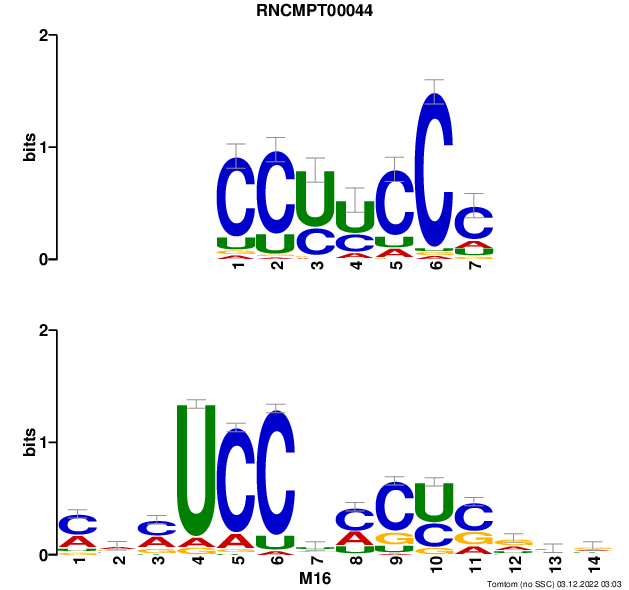 |
| i | j |
| 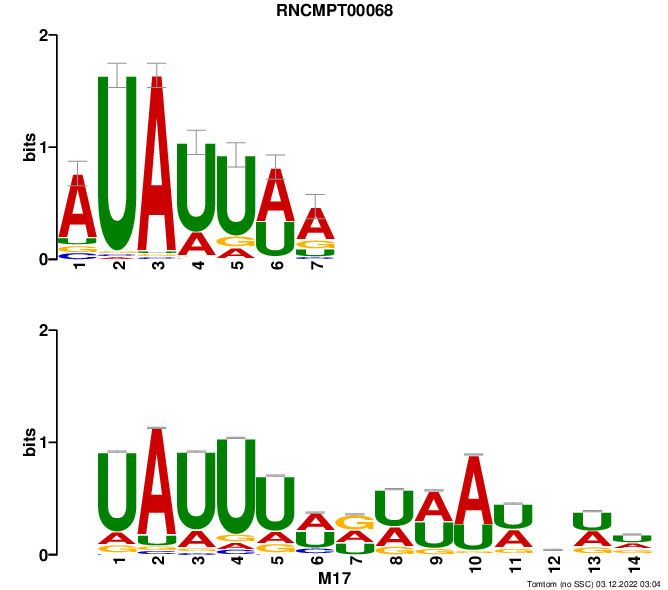 | 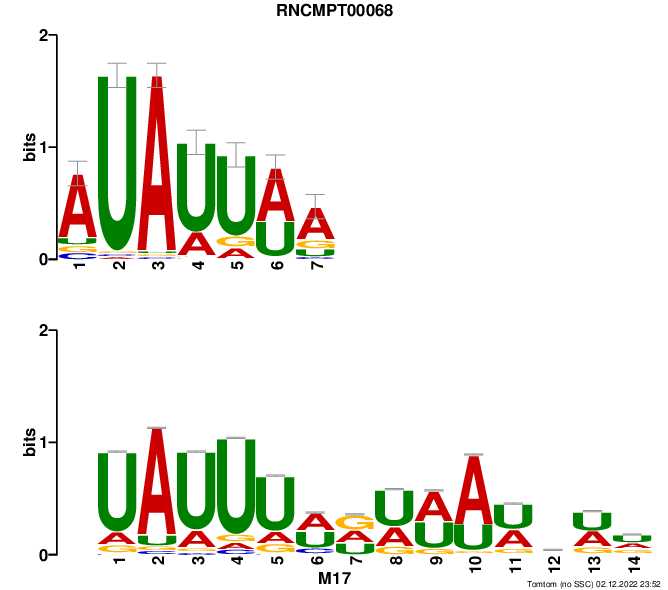 |
| k | l |
| 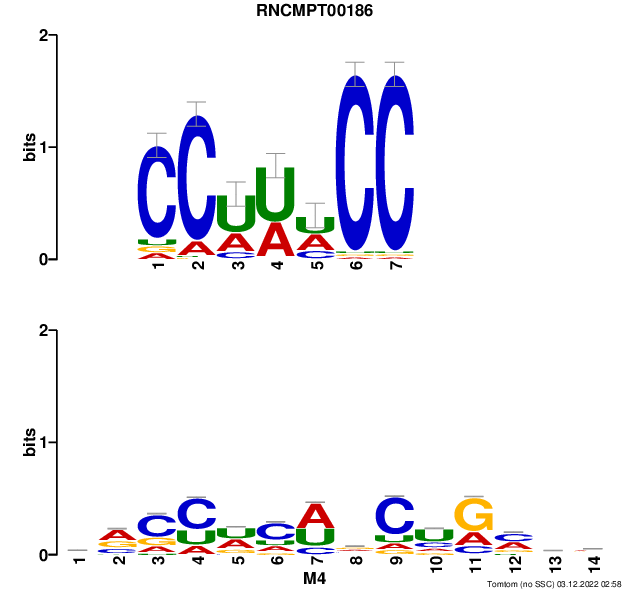 | 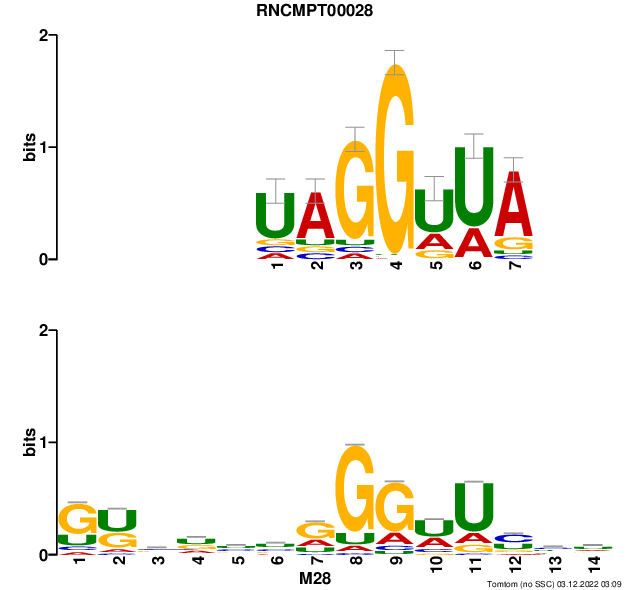 |
| m | n |
| 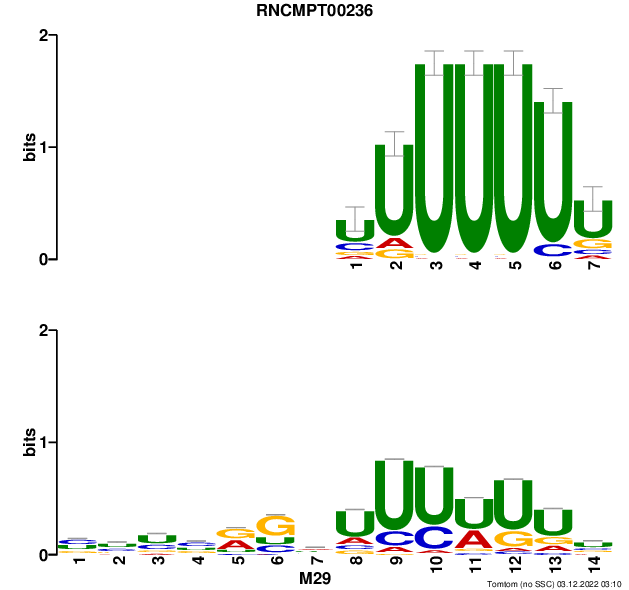 | 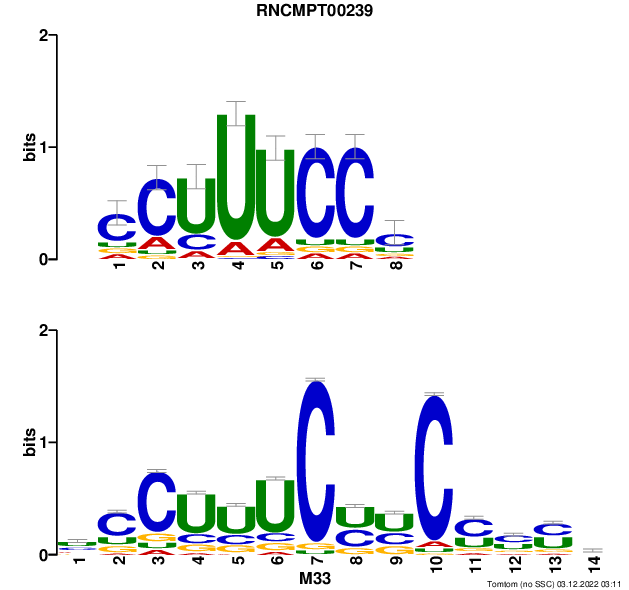 |
| o | p |
| 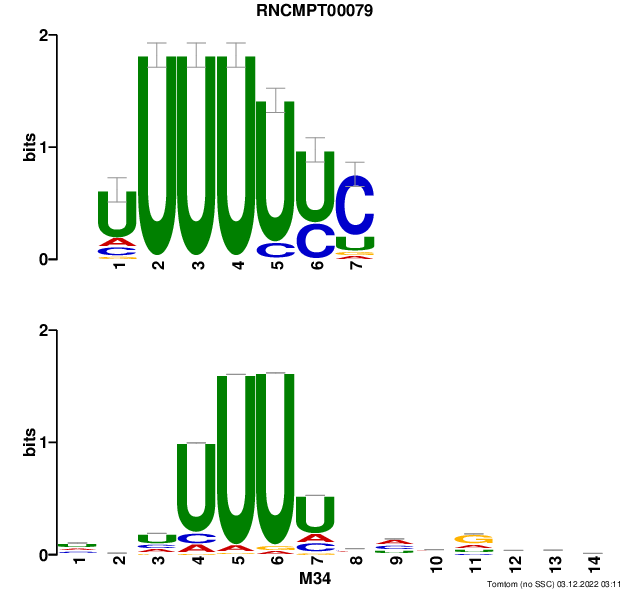 | 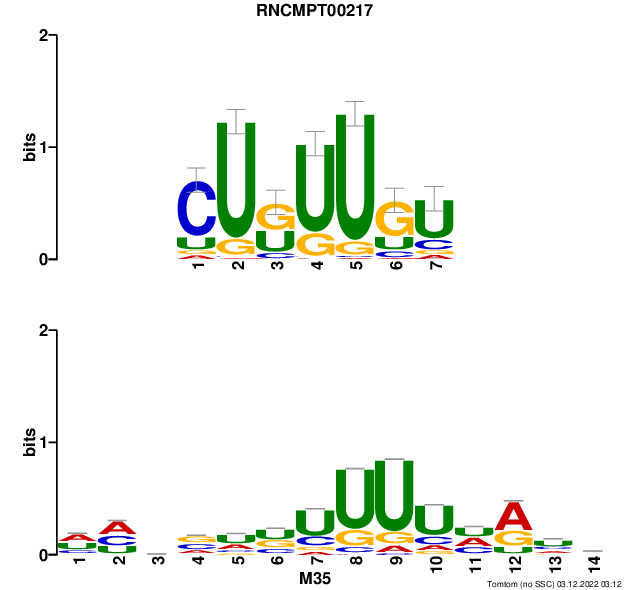 |
| q | r |
| 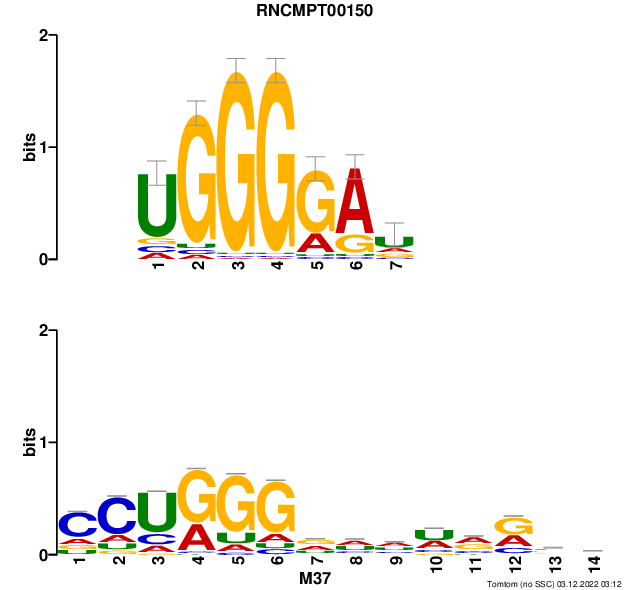 | 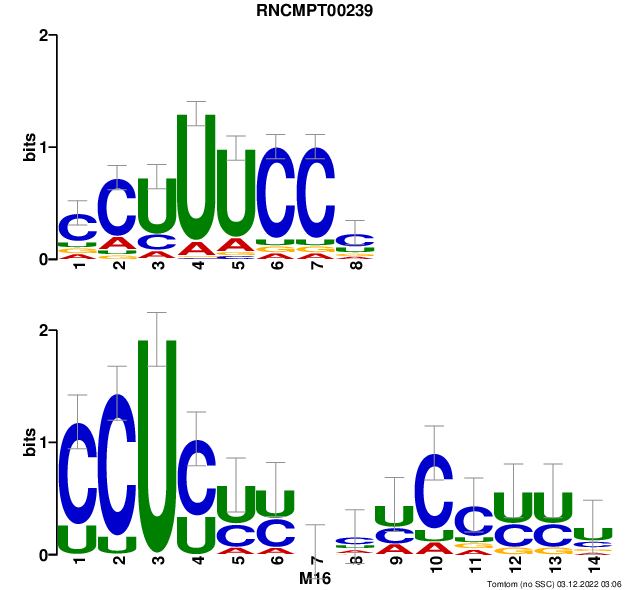 |
| s | t |
| 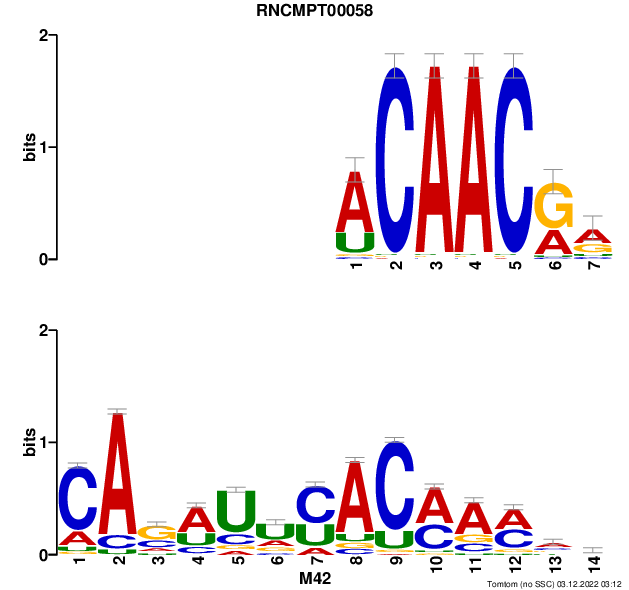 | 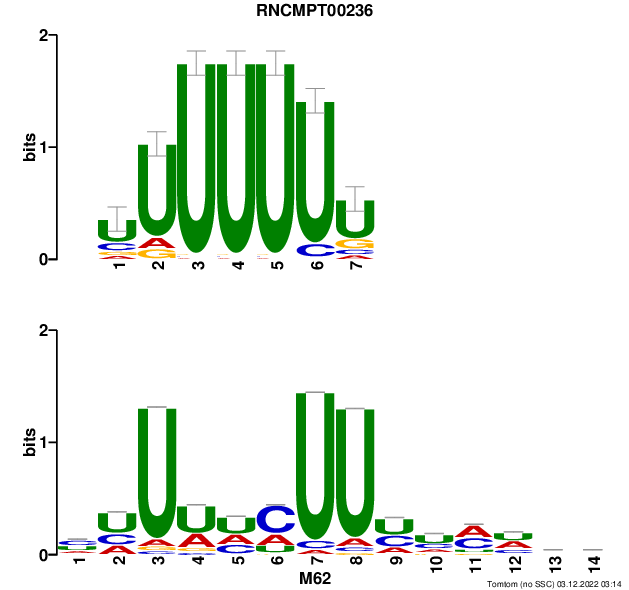 |
| u | v |
| 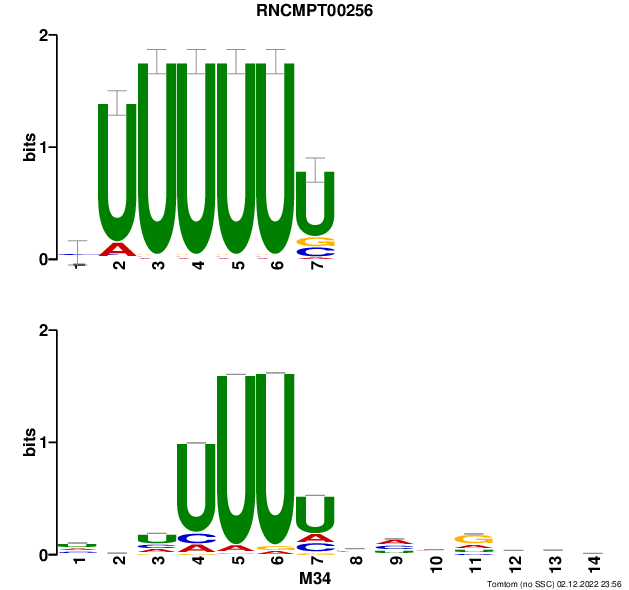 | 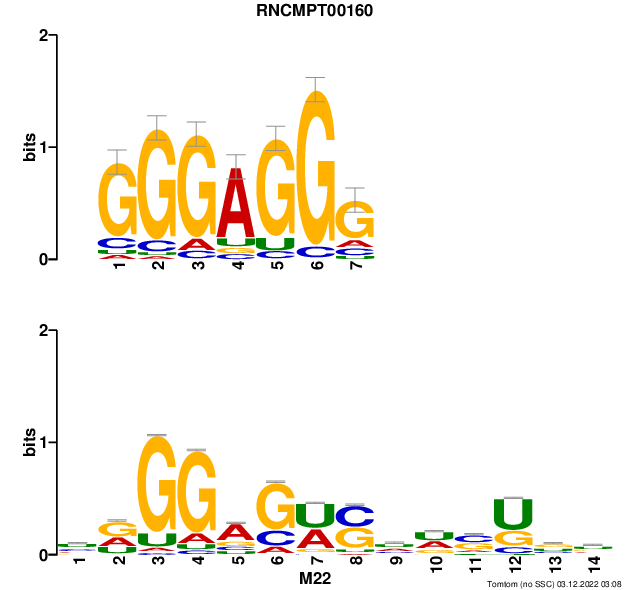 |
| w | x |

Fig. S 8 Extracted RNA motifs, as compared to the known motifs, using the TOMTOM tool with KDeep for jaspar dataset; a to e for experience 2 for RBP-24 datasets, f to m for experience 4 of RBP-31 datasets, n to x for experience 20 of RBP-31 datasets

## KDeep result for DNA dataset

Some of patterns found by KDeep with TomTom evaluation are shown in Fig. S 9. All patterns found are also in the KDeep_SM.zip file.

| 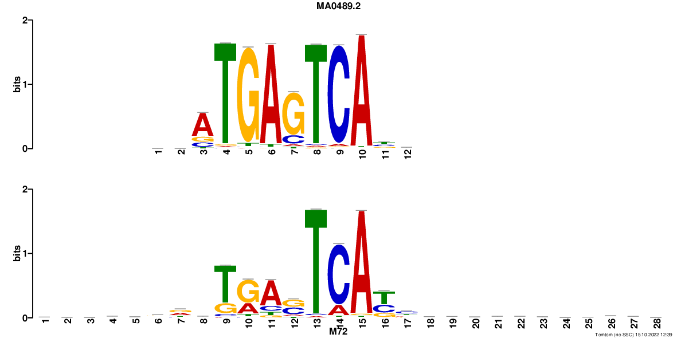 | 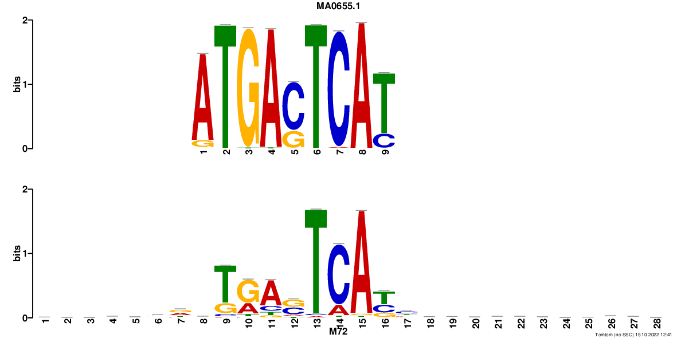 |
| --- | --- |
| a | b |
| 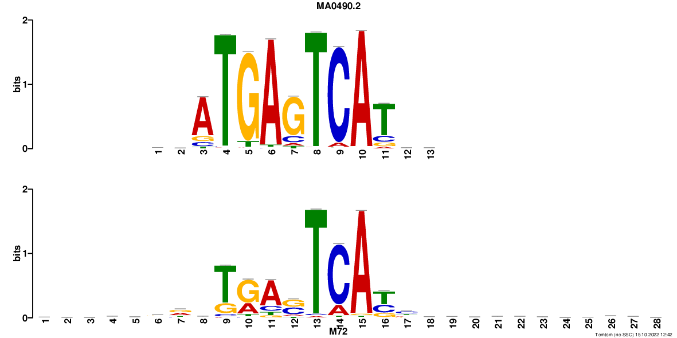 | 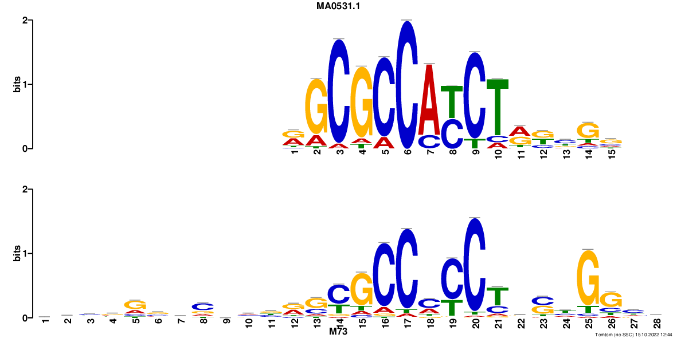 |
| c | d |
| 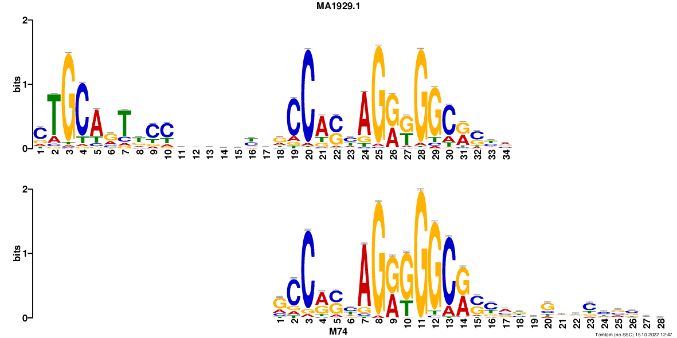 | 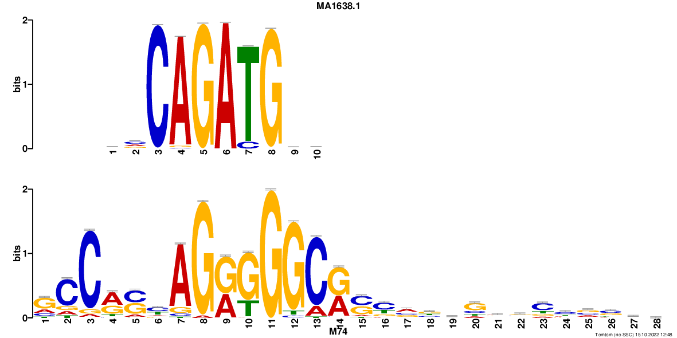 |
| e | f |
| 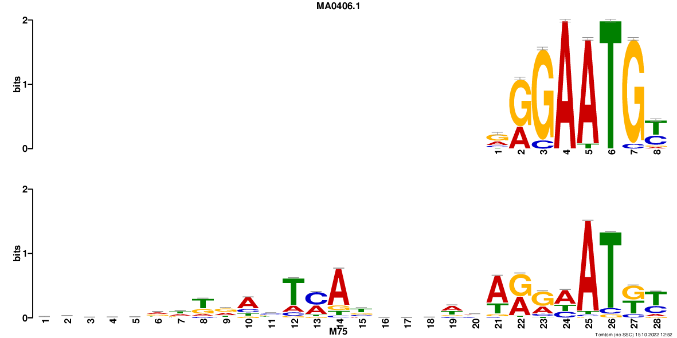 | 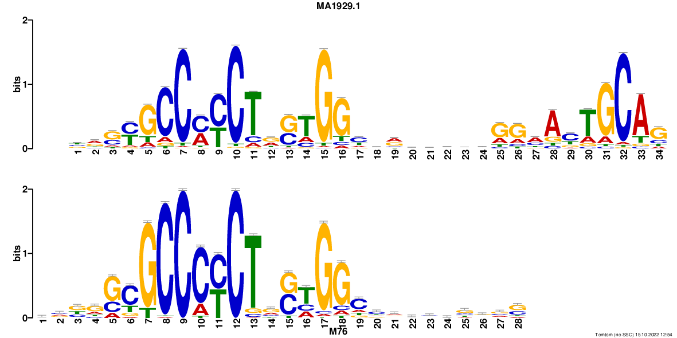 |
| g | h |
| 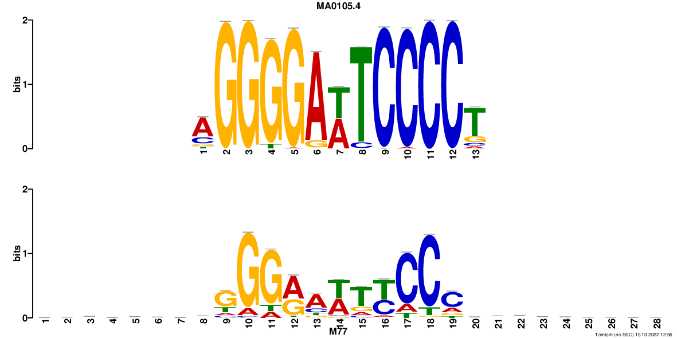 | 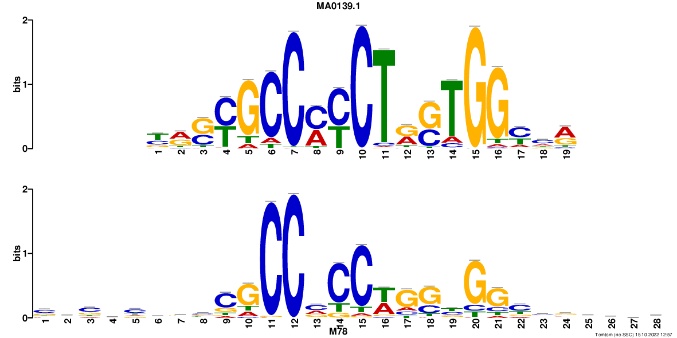 |
| i | j |
| 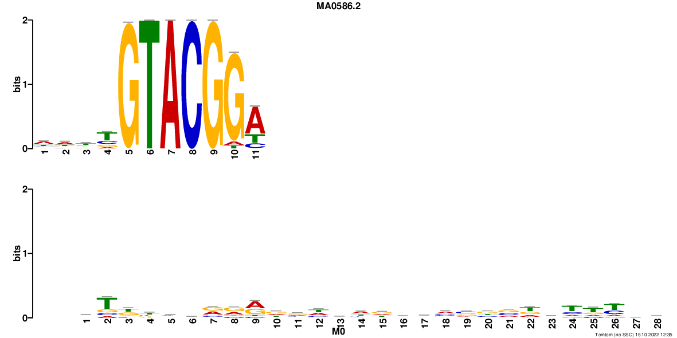 | |
| k | |

Fig. S 9 Extracted DNA motifs, as compared to the known motifs, using the TOMTOM tool with KDeep for jaspar dataset

## KDeep+ result for DNA dataset

Some of patterns found by KDeep+ with TomTom evaluation are shown in Fig. S 10. All patterns found are also in the KDeep_SM.zip file.

| 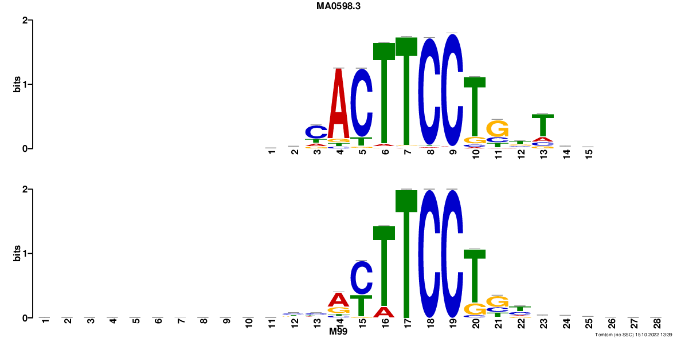 | 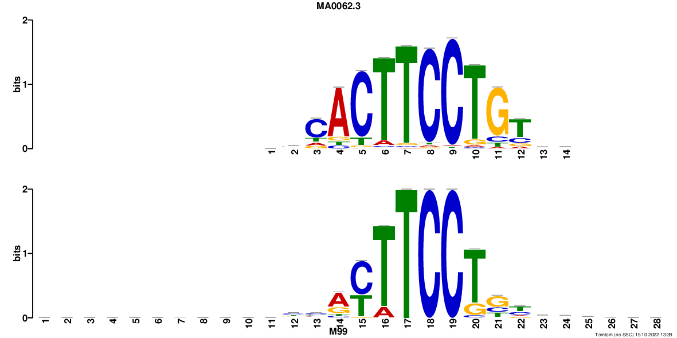 |
| --- | --- |
| a | b |
| 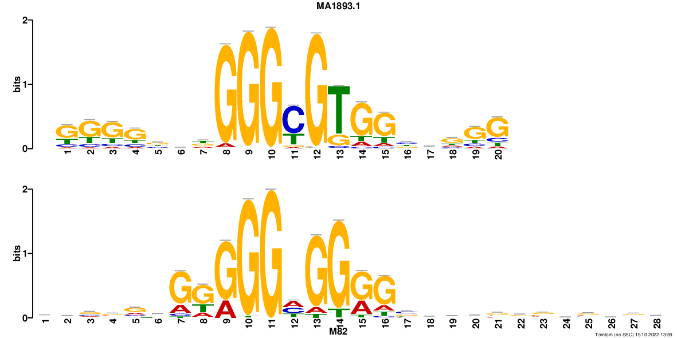 | 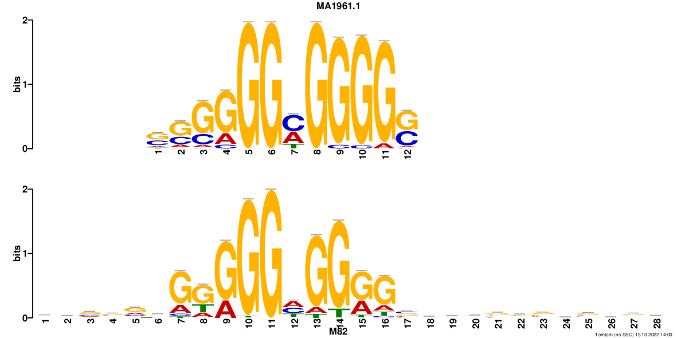 |
| c | d |
| 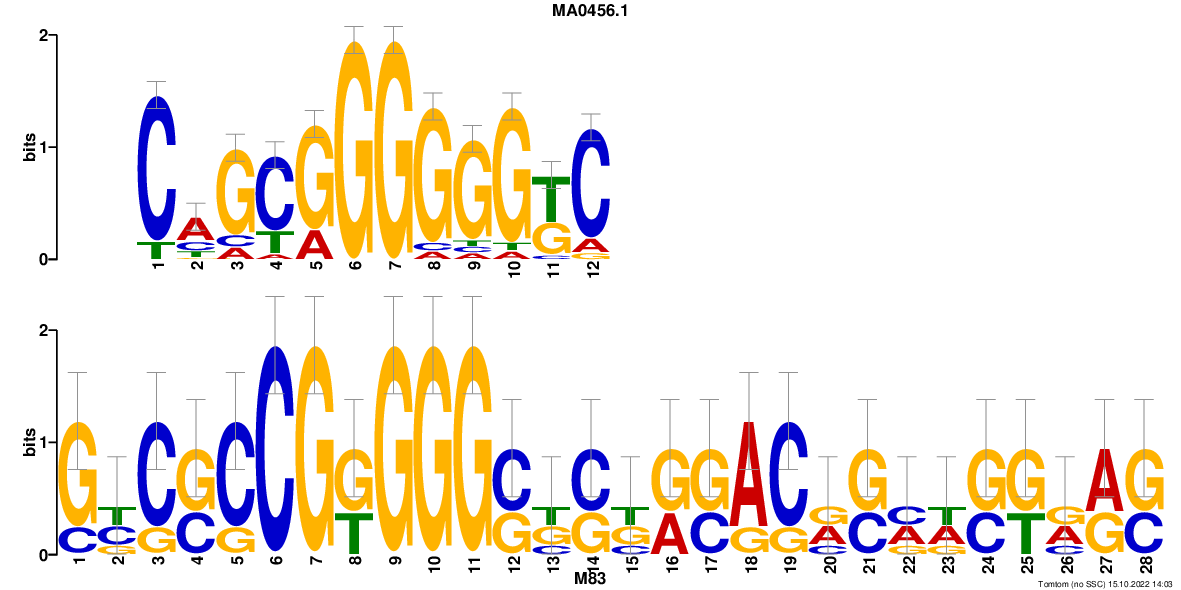 | 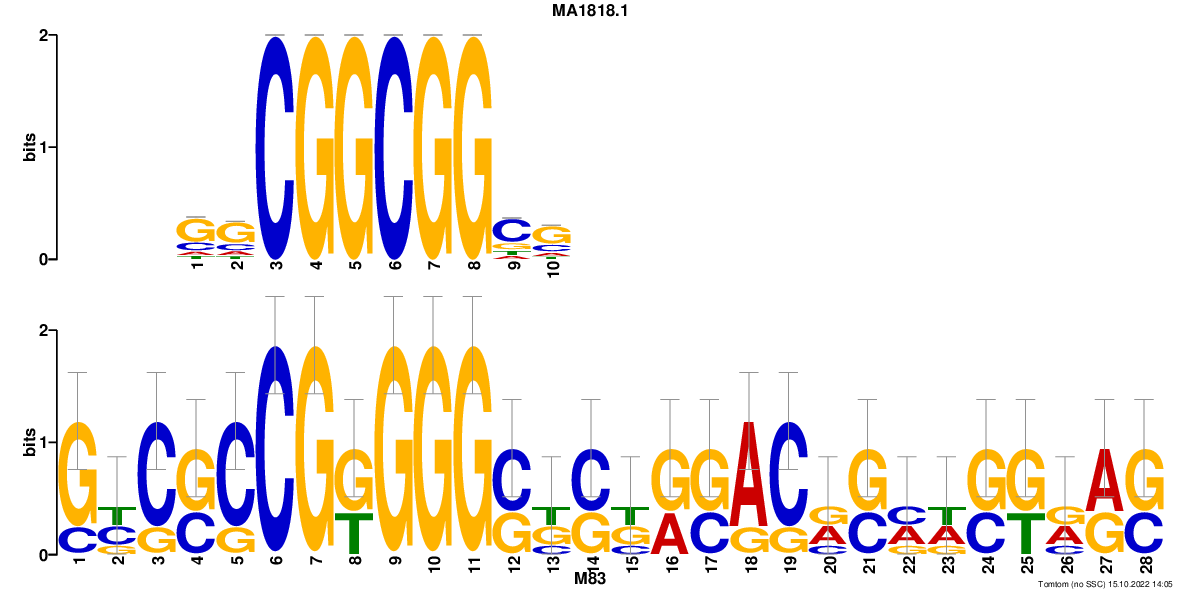 |
| e | f |
| 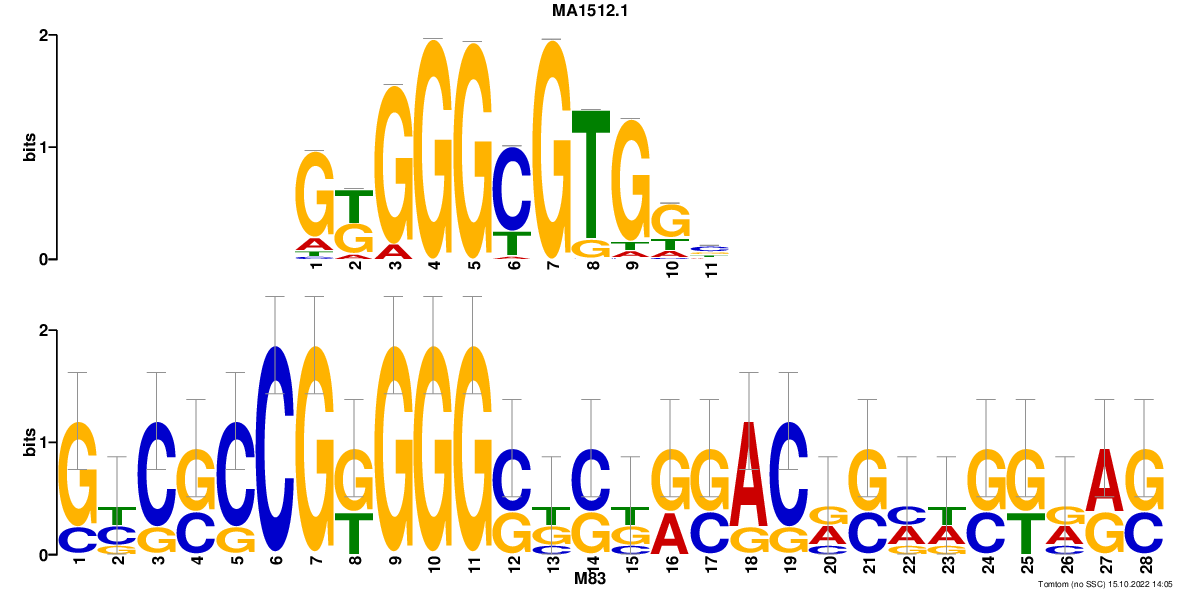 | 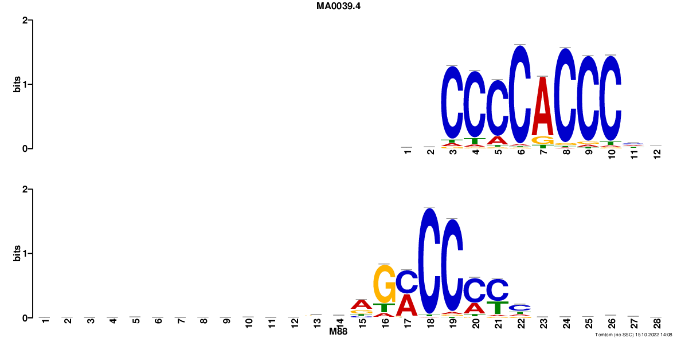 |
| g | h |
| 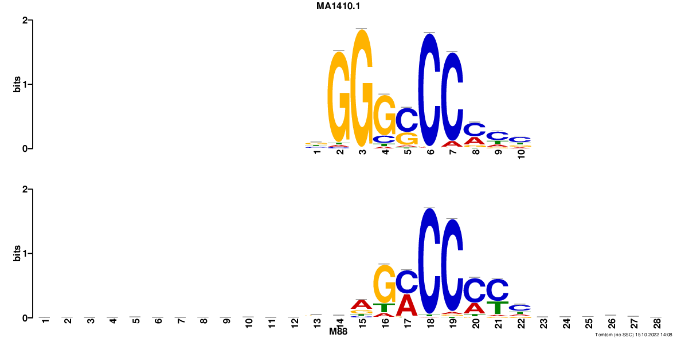 | 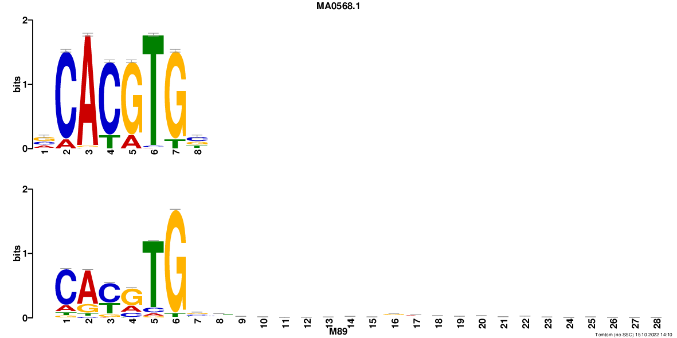 |
| i | j |
| 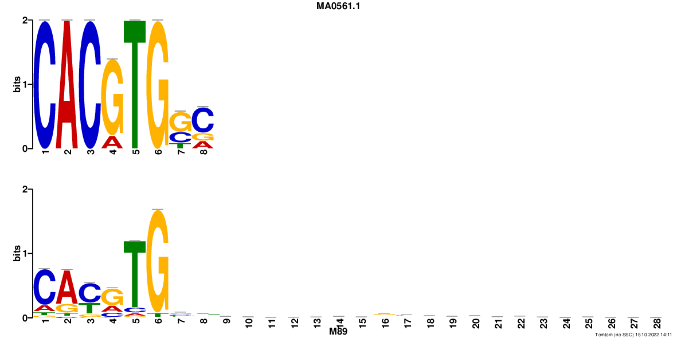 | 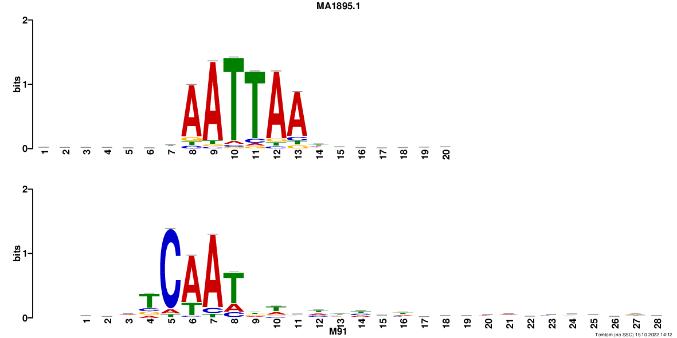 |
| k | l |
| 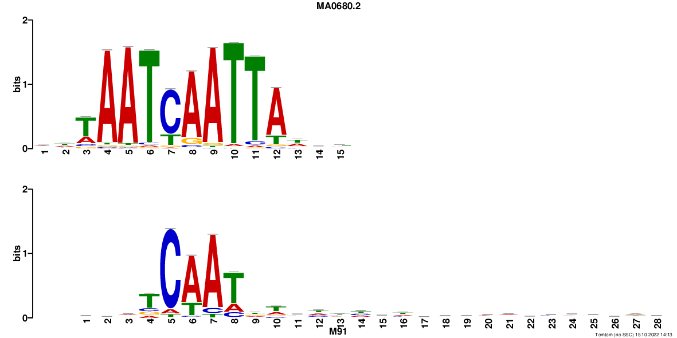 | 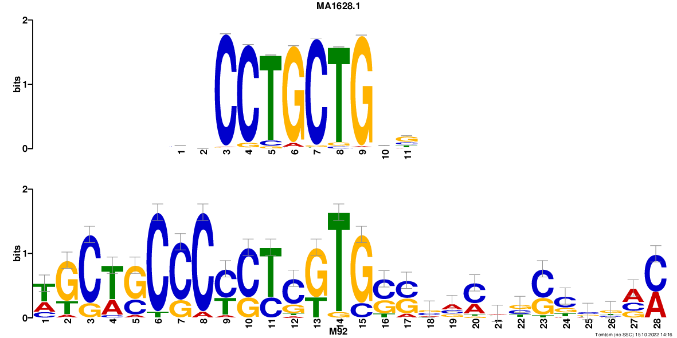 |
| m | n |
| 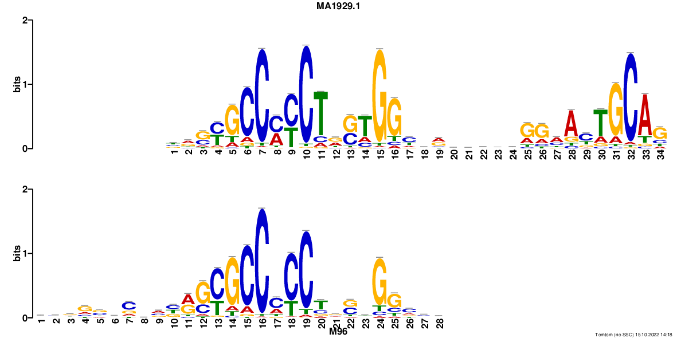 | 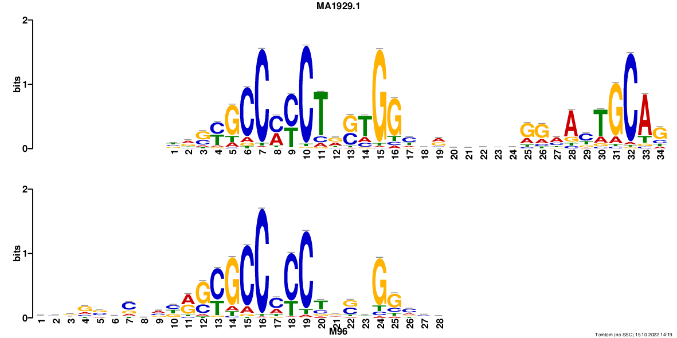 |
| o | p |
| 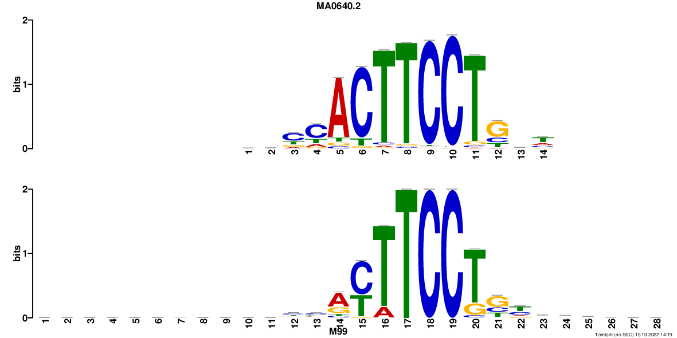 | 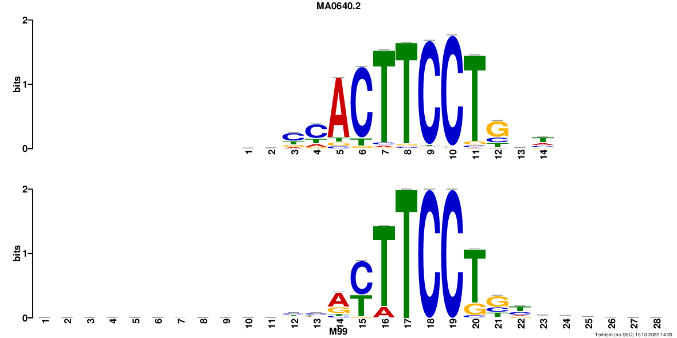 |
| q | r |
| 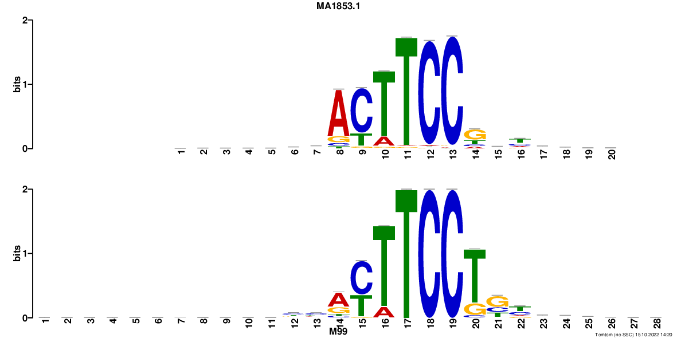 | 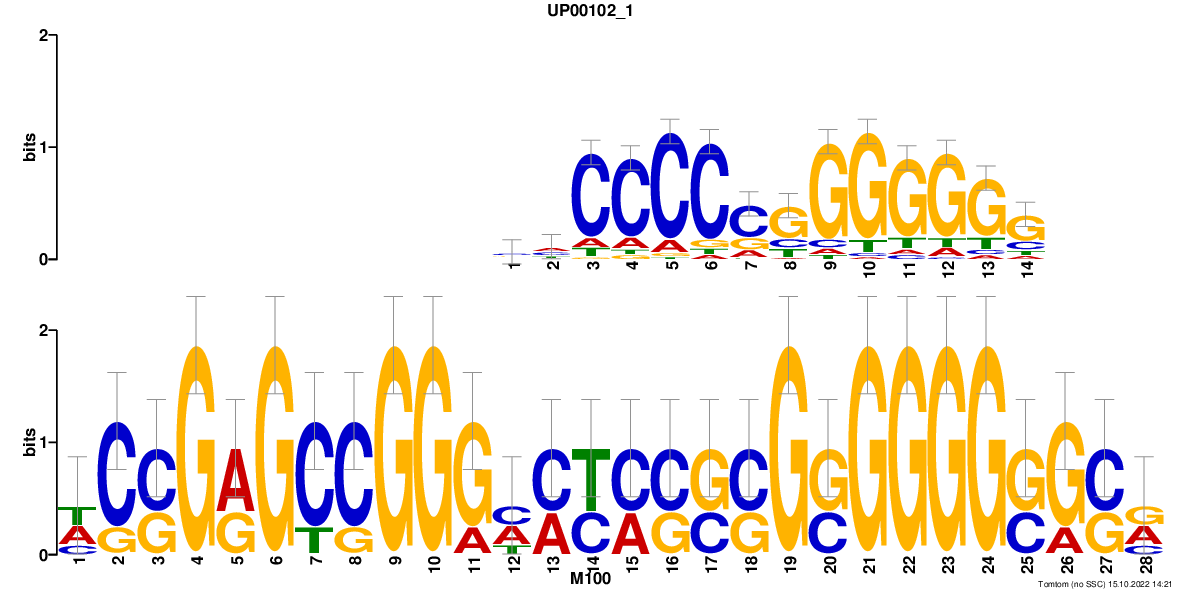 |
| s | t |
| 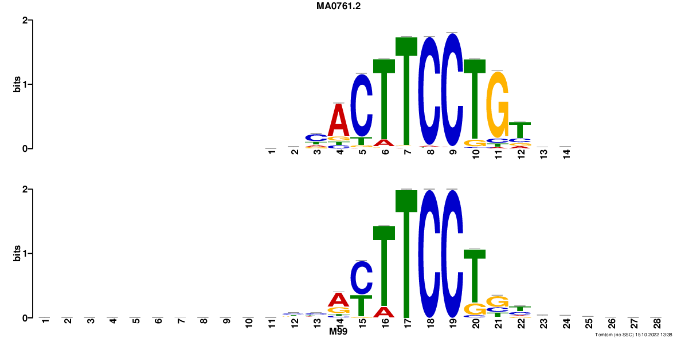 | |
| u | |

Fig. S 10 Extracted DNA motifs, as compared to the known motifs, using the TOMTOM tool with KDeep+ for jaspar dataset

| 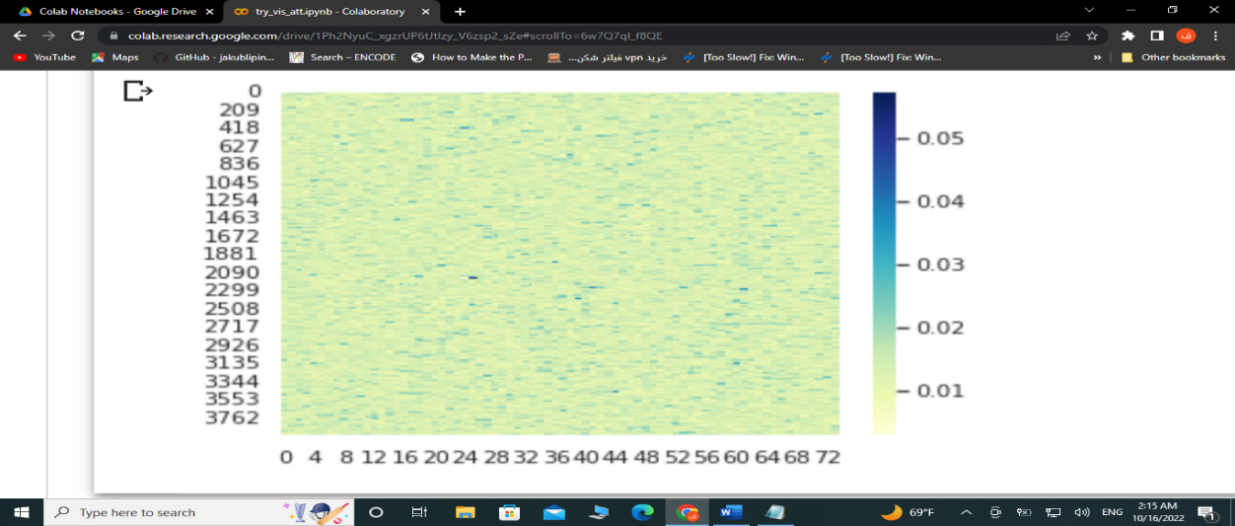 | 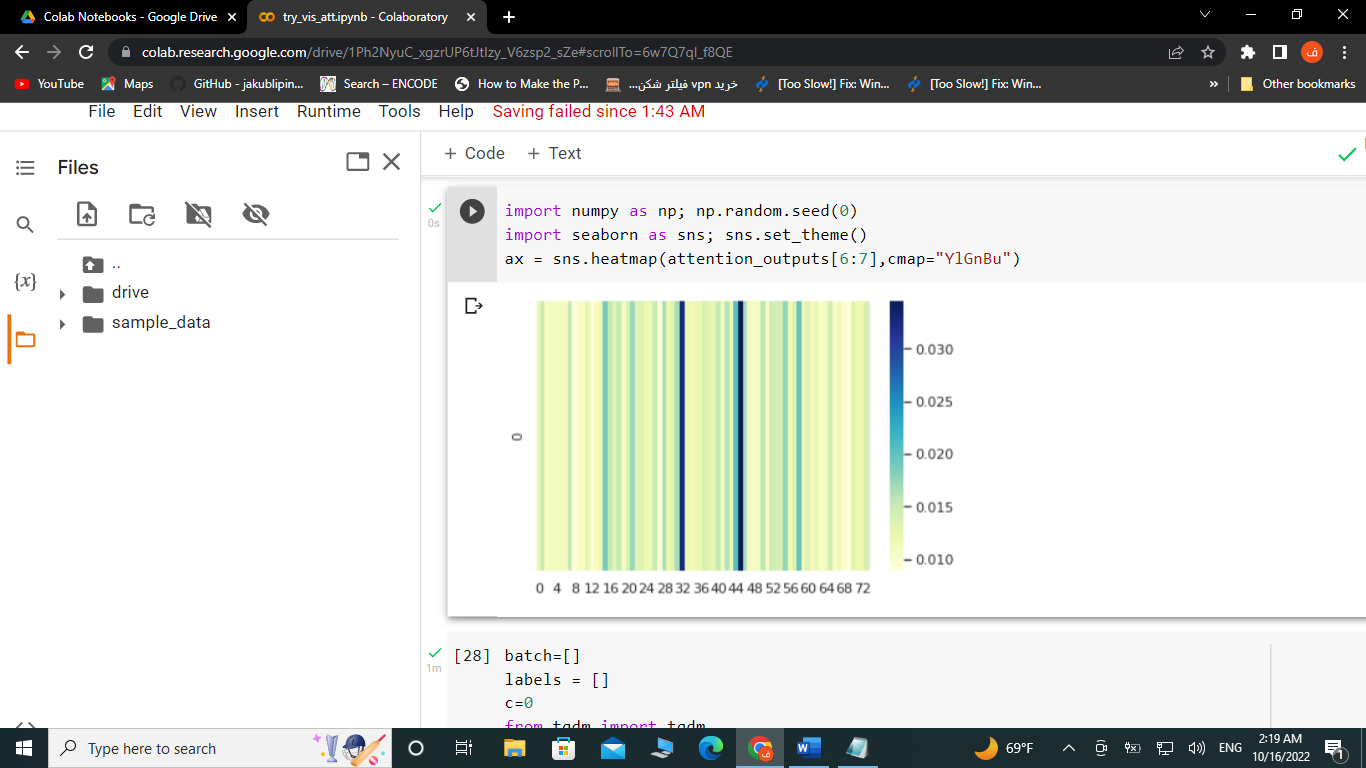 |
| --- | --- |
| **a** | **b** |

Fig. S 11 Heatmap of output score of attention layer in KDeep+ for DNA. a) Heatmap is related to all the samples that have at least one of the 14 (399, 383, 382, 377, 373, 351, 314, 313, 392, 269, 223, 184) TF type labels positive. b) Heatmap is related to one sample that have at least one of the 14 (399, 383, 382, 377, 373, 351, 314, 313, 292, 269, 223, 184) TF type labels positive and it shows that the middle of this sample has the ability of protein binding.

# References

[1] J. Zhou and O. G. Troyanskaya, “Predicting effects of noncoding variants with deep learning–based sequence model,” *Nat Methods*, vol. 12, no. 10, pp. 931–934, 2015, doi: 10.1038/nmeth.3547.

[2] K. Blin, C. Dieterich, R. Wurmus, N. Rajewsky, M. Landthaler, and A. Akalin, “DoRiNA 2.0—upgrading the doRiNA database of RNA interactions in post-transcriptional regulation,” *Nucleic Acids Res*, vol. 43, no. D1, pp. D160–D167, Jan. 2015, doi: 10.1093/nar/gku1180.

[3] M. Stražar, M. Žitnik, B. Zupan, J. Ule, and T. Curk, “Orthogonal matrix factorization enables integrative analysis of multiple RNA  binding proteins.,” *Bioinformatics*, vol. 32, no. 10, pp. 1527–1535, May 2016, doi: 10.1093/bioinformatics/btw003.

[4] A. Trabelsi, M. Chaabane, and A. Ben-Hur, “Comprehensive evaluation of deep learning architectures for prediction of DNA/RNA sequence binding specificities,” *Bioinformatics*, vol. 35, no. 14, pp. i269–i277, Jul. 2019, doi: 10.1093/BIOINFORMATICS/BTZ339.

[5] D. Maticzka, S. J. Lange, F. Costa, and R. Backofen, “GraphProt: modeling binding preferences of RNA-binding proteins,” *Genome Biol*, vol. 15, no. 1, p. R17, 2014, doi: 10.1186/gb-2014-15-1-r17.
